# FIERCE: reconstructing dynamic trajectories from the differentiation potency of single cells

**DOI:** 10.1093/bioinformatics/btag516

**Published:** 2026-07-13

**Authors:** Luca Calderoni, Oriana Romano, Francesco Grandi, Silvio Bicciato, Mattia Forcato

**Affiliations:** Department of Molecular Medicine, University of Padova, 35131 Padova, Italy; Department of Molecular Medicine, University of Padova, 35131 Padova, Italy; Department of Life Sciences, Centre for Genome Research, University of Modena and Reggio Emilia, 41125 Modena, Italy; Department of Molecular Medicine, University of Padova, 35131 Padova, Italy; Department of Molecular Medicine, University of Padova, 35131 Padova, Italy

## Abstract

**Motivation:**

Since the introduction of single-cell RNA sequencing (scRNA-seq), numerous computational approaches have been developed to reconstruct dynamic cellular processes from static transcriptional profiles. These methods order cells along continuous trajectories by assessing their similarity in the gene-expression space. However, they rely on several assumptions, such as prior knowledge of the structure and directionality of the expected genealogy. These assumptions can limit their application to complex cellular systems with poorly understood developmental paths.

**Results:**

To address this challenge, we introduce FIERCE (Framework for InfERence of the veloCity of Entropy), a novel computational pipeline designed to predict the changes in the differentiation potency of single cells during dynamic processes. Through a fully unsupervised approach, FIERCE enables the inference of cell lineages directly on the differentiation landscape of the biological system, thus eliminating the need for prior specification of developmental parameters. We demonstrate the efficacy of FIERCE by reconstructing three well-known mouse differentiation systems and by quantifying its accuracy on simulated data.

**Availability and implementation:**

The FIERCE R package is available on GitHub at https://github.com/bicciatolab/FIERCE

## 1 Introduction

The advent of high-throughput single-cell RNA sequencing (scRNA-seq) technologies has profoundly revolutionized transcriptomic studies, enabling researchers to explore the complete transcriptional profiles of individual cells instead of analyzing mixed gene expression patterns from entire tissues (Tang *et al.* 2009). This shift in perspective required the development of innovative concepts and paradigms to untangle the biological complexity embedded in these data (Luecken and Theis 2019). Among the various computational approaches designed for the analysis of scRNA-seq data, there has been a remarkable proliferation of methods aimed at reconstructing dynamic processes and developmental trajectories, with dozens of algorithms currently available to the scientific community (Chen *et al.* 2018, Saelens *et al.* 2019, Deconinck *et al.* 2021). Although these methods adopt a wide variety of computational frameworks, the most popular approach is grounded in a simple yet effective rationale. It is assumed that the biological sample comprises the full sequence of all cell states constituting its developmental history, and that, along this sequence, key genes involved in the process gradually change their expression levels from one state to the next. This allows cells to be ordered on a continuous trajectory based on their transcriptional similarity, yielding a so-called *pseudotemporal* sequence (Chen *et al.* 2018, Herring *et al.* 2018, Tritschler *et al.* 2019, Deconinck *et al.* 2021, Teschendorff and Feinberg 2021). Computationally, this is achieved by projecting cells onto a shared state manifold that reflects their relative distances in the gene-expression space. A proper root state is then set to compute a *pseudotime score*, defining cell progression on this manifold. In classic developmental processes, embryonic cells gradually lose their differentiation potency as they convert into more specialized states. Thus, the *pseudotime score* can be interpreted as an inverse proxy for such potency. Since this computational workflow begins at the whole-population level with the construction of the state manifold and then progresses to the single-cell level with the computation of the *pseudotime score* for each cell, it is termed a *top-down* approach (Teschendorff and Feinberg 2021). The primary drawback of the *top-down* approach is the need to establish the direction of the expected genealogical sequence by specifying a root state (Teschendorff and Feinberg 2021). This is often a delicate operation that can significantly influence the final trajectory. Since the state manifold is intrinsically undirected, it provides no information about the path each cell is currently taking along the dynamic process. Therefore, a directional component must be superimposed based on external knowledge. However, in lesser-known biological systems, it is often unclear, if not entirely unknown, which subpopulation should serve as the root state.

To address this significant limitation, one can adopt an alternative, bottom-up approach that directly reconstructs the final trajectory from the dynamic changes in the differentiation potency of each cell during a dynamic process (Teschendorff and Feinberg 2021). Inspired by the principles of statistical mechanics, this strategy aims to reconstruct the behavior of macroscopic dynamic processes by analyzing the dynamics of all their microscopic components, namely, the single cells (MacArthur and Lemischka 2013, Teschendorff and Feinberg 2021). To the best of our knowledge, despite the wide variety of available trajectory reconstruction methods, a coherent framework entirely designed according to a potency-based *bottom-up* approach is still missing. Recent studies have demonstrated that the transcriptomes of cells contain a significant amount of information that can be leveraged to infer their differentiation potencies (Banerji *et al.* 2013, Guo *et al.* 2017, Teschendorff and Enver 2017, Chen *et al.* 2019). The rationale behind this is that as cells differentiate into increasingly specialized states, they tend to concentrate their gene expression signals into more specific molecular pathways. Therefore, it becomes possible to estimate their potencies by quantifying the level of dispersion of their expression signals across various pathways, e.g. by computing their transcriptional entropy (Banerji *et al.* 2013, MacArthur and Lemischka 2013, Guo *et al.* 2017, Teschendorff and Enver 2017, Chen *et al.* 2019). However, the entropy scores alone do not inherently provide any information about the trajectory direction, which must once again superimposed by the user as in the *top-down* approach. Indeed, only if we have reason to assume that a dynamic process adheres to the principles of classical development can we safely expect the entropy of cells to gradually decrease from pluripotent or multipotent states to fully differentiated states (Guo *et al.* 2017, Teschendorff and Enver 2017, Chen *et al.* 2019, Gulati *et al.* 2020). In the absence of such an assumption, there is currently no way to infer the evolution over time of cell potencies in a completely unsupervised manner.

Here, we aim to fill this gap by introducing FIERCE (Framework for InfERence of veloCity of Entropy), a novel trajectory reconstruction method entirely inspired by the *bottom-up* approach. FIERCE reconstructs trajectories from scRNA-seq data by predicting the future dynamics of transcriptional entropies in single cells, and thus does not require any prior knowledge of the biological system under study. To demonstrate the potential of our algorithm, we applied FIERCE to the reconstruction of three well-known mouse developmental processes and proved its capability to identify the correct trajectories without being provided with any prior information. Moreover, we show the additional insights that FIERCE offers compared to traditional *top-down* methods in such unsupervised conditions. Based on our findings, we envisage that FIERCE will be a valuable tool for reconstructing the developmental history of lesser-known biological systems and will contribute to a new *bottom-up* philosophy in trajectory reconstruction that aligns more closely with the reductionist perspective provided by single-cell sequencing data.

## 2 Materials and methods

### 2.1 FIERCE

#### 2.1.1 Computational details

FIERCE is a computational pipeline designed to reconstruct both the structure and dynamics of the differentiation potency landscape of cell populations from scRNA-seq data. To achieve this task, FIERCE integrates the inference of cell differentiation potency based on signaling entropy (Teschendorff and Enver 2017) with the reconstruction of dynamic changes in entropy scores using the information provided by RNA velocity (La Manno *et al.* 2018, Bergen *et al.* 2020). FIERCE operates in the R environment and calls specific Python functions and modules through the *reticulate* package (Ushey *et al.* 2024). The FIERCE pipeline consists of three main steps: (i) inferring the future transcriptional states of cells through RNA velocity, (ii) computing the velocity of the entropy of cells as the difference between their future and observed signaling entropy, and (iii) constructing the velocity of the entropy vector field and generating a *streamplot* that depicts the trajectories followed by cells on the differentiation potency landscape during the dynamic process.

#### 2.1.2 Inference of future transcriptional states

Future transcriptional states are predicted by exploiting an implication of the RNA velocity method. Since RNA velocity quantifies the change (e.g. increase or decrease) in the spliced counts of a gene over a time unit (La Manno *et al.* 2018, Bergen *et al.* 2020), by adding this change to the observed spliced counts, it is possible to predict the future spliced counts of the gene after one unit of time. Extending this process to all genes across all cells results in the complete future spliced gene expression matrix for the entire dataset.

In the first step of FIERCE, RNA velocity is estimated using the dynamical model implemented in the scVelo Python package (v0.2.4) (Bergen *et al.* 2020). The choice of scVelo is motivated by the fact that, differently from velocyto (La Manno *et al.* 2018) (i.e. its original implementation), the key dynamic parameters are not defined in units of splicing rate, allowing the estimation also for genes with insufficient cells in steady state. Moreover, the scVelo algorithm, by default, computes both dynamical model fitting and velocity on the first-order moments of spliced and unspliced transcript counts, rather than on the respective raw counts. On a precomputed neighborhood graph, the first-order moments of a cell’s transcript counts correspond to the gene-wise means across its nearest neighbors (30 by default in scVelo) (Bergen *et al.* 2020). This approach accentuates the dynamic trend of each gene across cells. The RNA velocity $v_{s}$ of gene i in cell j is therefore defined as the rate of change of its spliced first-order moments $M_{s}$:


(1) $$v_{s(ij)}={dM}_{s(ij)}/dt$$

FIERCE employs scVelo to quantify the RNA velocities of all genes in all cells from the respective spliced and unspliced first-order moments. Given that only a minor subset of genes exhibits a genuine dynamic behavior across cells in any biological process, the dynamical model will successfully fit only a minority of genes. For all other genes, the RNA velocity is set to 0 in all cells.

After computing velocities, FIERCE adds the matrix of RNA velocities for all genes across all cells to the matrix of the observed spliced first-order moments for all genes across all cells:


(2) $$M_{s}^{'}=M_{s}+v$$

This results in the matrix of predicted future spliced first-order moments, which represents the future transcriptional states. These states are then utilized to reconstruct the dynamics of cells at the potency level. All computations for this initial step are handled by the *compute_future_states* function.

#### 2.1.3 Computation of the velocity of the entropy

The second step of FIERCE involves: (i) computing both the observed and future signaling entropy scores of cells from their observed and future transcriptional profiles, respectively and (ii) calculating the velocity of the entropy of cells as the difference between their future and observed signaling entropy profiles.

Signaling entropy is a complex metric formulated to measure the transcriptional entropy (and, consequently, the differentiation potency) of cells from their scRNA-seq data (Teschendorff and Enver 2017). It is computed by the SCENT R package (v1.0.3) (Teschendorff and Enver 2017, Chen *et al.* 2019), which quantifies the dispersion of gene expression signals across all molecular pathways defined in a cell-shared genome-wide protein-protein interaction (PPI) network (Banerji *et al.* 2013). A crucial characteristic of signaling entropy is that it can be computed both for each cell, as the respective total signaling entropy score, and for each gene in each cell, as the respective local signaling entropy score, provided that the protein product of the gene is present within the PPI network.

To compute the signaling entropy scores of cells, FIERCE utilizes the SCENT algorithm with the spliced first-order moments as the expression signal. Specifically, for each cell, the signaling entropy is computed twice: first, from the observed moments ($M_{s}$), and then from the future moments ($M_{s}^{\prime }$). Both the total entropy score of each cell and the local entropy score of each gene in each cell are computed. This results in (i) two vectors containing the observed and future total signaling entropy scores of all cells and (ii) two new matrices containing the observed (e) and the future (e′) local signaling entropy scores of all genes in all cells. The *velocity of the entropy* $v_{e}$, which defines the dynamic movements of cells in the signaling entropy space, is computed by subtracting the matrix of observed local entropies from that of the future local entropies. This results in a matrix that represents, for each gene in each cell, the change in the local entropy score over a unit of time:


(3) $$v_{e}=e^{'}-e$$

The computation of signaling entropy involves only the genes in the dataset whose protein products are effectively present within the PPI network. Therefore, the computation of the velocity of the entropy, as well as all downstream operations related to the construction of the velocity of the entropy vector field, are executed exclusively on such genes.

FIERCE incorporates the original built-in human PPI network from SCENT (derived from the integration of several databases from the PathwayCommons platform (Cerami *et al.* 2011)) and four additional networks constructed *de novo* for human and mouse. These PPI networks have been created based on the protein-protein functional interactions stored in the STRING database (Snel *et al.* 2000, Szklarczyk *et al.* 2021). Following the guidelines provided by STRING (https://string-db.org/), we constructed, for both human and mouse, a *medium-confidence* network including all interactions with a minimum STRING score of 0.4 (human network includes 18 337 genes and 1 670 338 interactions; mouse network includes 19 395 genes and 1 699 484 interactions), and a *high-confidence* network including all interactions with a minimum STRING score of 0.7 (human network includes 16 058 genes and 471 618 interactions; mouse network includes 15 850 genes and 423 898 interactions). In FIERCE, the *high-confidence* network is used as the default for both human and mouse.

All the computations described above are performed by the *compute_signaling_entropy* function of FIERCE. Optionally, this function can also subdivide the cell population into *potency states* (i.e. into clusters that represent discrete stable states of the differentiation potency landscape). These are obtained by fitting a mixture of Gaussian curves to the overall distribution of total signaling entropy scores (Teschendorff and Enver 2017) and are numbered according to their median entropy score in reverse order (i.e. the first potency state has the highest median entropy and the last has the lowest). This approach provides an initial insight into the overall structure of the differentiation potency landscape of the population before a more detailed view is provided by the velocity of the entropy vector field.

#### 2.1.4 Construction of the vector field

The purpose of the velocity of the entropy is to predict the future movements of cells on a reduced dimensional space that encapsulates the structure of the differentiation potency landscape of the population. This prediction is accomplished through the construction of the velocity of the entropy vector field, which involves two separate computational steps. Firstly, a uniform manifold approximation and projection (UMAP) embedding is constructed directly from the observed local signaling entropy scores of all cells. Then, the future positions of all cells on this embedding are predicted based on their velocity of the entropy and the local entropy scores of their neighbors.

In the first step, a principal component analysis (PCA) is performed directly on the observed local entropies matrix e, and the resulting principal components are used to construct a neighborhood graph (30 neighbors for each cell by default) and a UMAP embedding. Both operations are handled by the *compute_entropy_UMAP* function of FIERCE, which exploits the Scanpy Python package (v1.9.1) (Wolf *et al.* 2018).

The second step involves an adaptation of the scVelo *velocity_graph* function, which is specifically designed to construct RNA velocity vector fields in the gene-expression space (Bergen *et al.* 2020). Specifically, a *velocity graph* π on the signaling entropy space is first constructed by calculating, for each cell i, a series of cosine correlations between the vector containing the velocity of the entropy ($\nu_{e}$) of all its genes and each vector containing, for the same genes, the difference (δ) between the local signaling entropy score of cell i and the local signaling entropy score of each of its neighbors j in the signaling entropy space:


(4) $$\delta_{ij}=e_{j}-e_{i}$$

(5) $$\pi_{ij}=\text{cos}\;∠(\delta_{ij};\nu_{e_{i}})=\frac{\delta_{ij}^{T}\nu_{e_{i}}}{\left\|\delta_{ij}\|\|\nu_{e_{i}}\right\|}$$

The neighbors j are identified through a recursive neighbor search on the neighborhood graph computed in the previous step (with the default of 30 neighbors, this results in 100–200 potential transitions) (Bergen *et al.* 2020). During the construction of the velocity graph, a variance-stabilizing transformation is performed by default. This involves computing the cosine correlations between $\mathrm{sign}(\delta_{ij})\sqrt{\left|\delta_{ij}\right|}$ and $\mathrm{sign}(\nu_{e_{i}})\sqrt{\left|\nu_{e_{i}}\right|}\;$to obtain clearer and smoother vector fields (Bergen *et al.* 2020). Then, the velocity graph is converted into a cell-cell transition probability matrix through an exponential kernel:


(6) $${\tilde{\pi }}_{ij}=\frac{1}{z_{i}}e\mathrm{xp}(\frac{\pi_{ij}}{\sigma_{i}^{2}})$$

where $z_{i}=\sum_{j}\;\text{exp}\;(\frac{\pi_{ij}}{\sigma_{i}^{2}})$ is a row normalization factor and $\sigma_{i}$ is the kernel width parameter (0.1 by default) (Bergen *et al.* 2020).

This cell-cell transition probability matrix defines the probability of each cell to move toward each of its neighbors in the signaling entropy space based on the velocity of the entropy of its genes. The positions of cells on the entropy-derived UMAP embedding, computed in the previous step, are defined by a set of coordinate vectors ${\tilde{e}}_{1}$, …, ${\tilde{e}}_{n}$. Therefore, the vectors of two cells i and j can be subtracted from one another to obtain the distance between their respective positions on the embedding:


(7) $${\tilde{\delta }}_{ij}=\frac{{\tilde{e}}_{j}-{\tilde{e}}_{i}}{\left\|{\tilde{e}}_{j}-{\tilde{e}}_{i}\right\|}$$

The future position of cell i in the embedding is defined as its displacement from its current position, also referred to as *embedded velocity* (${\tilde{\upsilon }}_{i}$) (Bergen *et al.* 2020). This displacement is computed by weighting the distances ${\tilde{\delta }}_{ij}$ of cell i from all its neighbors j based on the respective transition probabilities ${\tilde{\pi }}_{ij}$:


(8) $${\tilde{\upsilon }}_{i}=\sum_{j\neq i}w_{ij}{\tilde{\delta }}_{ij}=\sum_{j\neq i}({\tilde{\pi }}_{ij}-\frac{1}{n}){\tilde{\delta }}_{ij}$$

where subtracting $\frac{1}{n}$ is used to correct for the non-uniform density of points in the embedding. Optionally, the neighborhood graph used for computing the velocity graph and embedded velocities can differ from the graph used for computing the entropy-derived UMAP embedding in the previous step. This decoupling enables the computation of transition probabilities and coordinate displacements of cells over a specific number of neighbors j without the need to change and recompute the underlying UMAP embedding.

Once the embedded velocities have been computed for all cells, an arrow is drawn to connect the current position of each cell to its predicted future position. Specifically, FIERCE utilizes the scVelo *velocity_embedding_stream* function, which is designed to first draw the vector field on a grid, and then to merge the arrows of nearby grid points into continuous curves, enhancing the visualization of the overall dynamics of the cell population (Bergen *et al.* 2020). The final *streamplot* yielded by this procedure provides, in a single image, a comprehensive picture of both the structure and the predicted dynamic change of the differentiation potency landscape of the biological system. The entire computational procedure involving the construction of the velocity of the entropy vector field is performed by the *compute_graph_and_stream* function of FIERCE.

### 2.2 Pancreas endocrinogenesis dataset

#### 2.2.1 Preprocessing

The pancreas endocrinogenesis dataset (Bastidas-Ponce *et al.* 2019) was retrieved from the scVelo Python package (v0.2.4), where it is stored as a test dataset. The expression data (obtained using the 10X Chromium technology) include both spliced and unspliced raw counts for a total of 27 998 genes in a subset of 3696 cells from embryonic Day 15.5 of the dataset published by Bastidas-Ponce *et al.* (2019) (Table 1, available as supplementary data at *Bioinformatics* online). Additionally, the scVelo test dataset comprises 50 principal components computed from 4004 highly variable genes, along with the UMAP coordinates computed from these components. Such precomputed data were used to start the FIERCE analysis.

#### 2.2.2 FIERCE analysis

We started the FIERCE analysis by constructing a neighborhood graph with 30 neighbors per cell using the *perform_preprocessing* function on the first 30 principal components. Subsequently, we input the neighborhood graph to the *compute_future_states* function to compute the first-order moments of spliced and unspliced counts. The signaling entropy was quantified using FIERCE’s built-in *high-confidence* murine PPI network, resulting in 14 789 genes being retained from the original dataset. To construct the entropy-derived UMAP embedding, we computed 50 principal components from the observed local entropy scores and utilized all such components to construct a neighborhood graph with 30 neighbors for each cell. This graph was also employed during the vector field construction to compute the velocity graph and the embedded velocities.

### 2.3 Mammary gland development dataset

#### 2.3.1 Preprocessing

We downloaded both raw UMI counts from Gene Expression Omnibus (GEO, GSE111113) and original fastq files from the Sequence Read Archive (SRA, SRP133477) of all four samples included in the murine mammary gland (MG) development dataset produced with the 10X Chromium technology by Giraddi *et al.* (2018). The samples were sequenced at four different time points: embryonic Day 16, embryonic Day 18, postnatal Day 4, and postnatal Week 12 (adult). Expression data, consisting of 19 105 genes profiled across a total of 6784 cells, were imported in R and analyzed using Seurat (v3.1.5) (Stuart *et al.* 2019) R package. Low-quality cells were identified as outliers within the distribution of the number of genes, UMI counts, and fraction of counts mapping to mitochondrial genes per cell, and subsequently discarded. Multiplet cells were detected using the Scrublet Python package (v0.2.1) (Wolock *et al.* 2019). The final dataset consisted of 5669 cells. Prior to dimensional reduction with PCA, we log-normalized the data with a scale factor of 10 000 assigned a cell cycle score to each cell, computed 2000 highly variable genes with the *vst* procedure, and scaled the data. We assigned cell type identities using the scMCA R package (v0.2.0) (Han *et al.* 2018, Fei *et al.* 2022, Wang *et al.* 2023) and then filtered each sample to retain only cells assigned to mammary epithelial types, thus resulting in 5168 total cells (Table 2, available as supplementary data at *Bioinformatics* online). We then merged the four samples using Seurat (v3.1.5) data integration procedure. Before dimensional reduction with PCA of the integrated dataset, we performed regression using the cell cycle scores. We selected 18 principal components to compute the UMAP embedding. The principal components, cell annotations, and UMAP coordinates were successively transferred from the integrated Seurat object to the *anndata* object used for the FIERCE analysis. This object was created as follows. We initially utilized Cellranger (v3.1.0) (Zheng *et al.* 2017) to align the reads contained in the fastq files of each single sample to the reference mm10 mouse genome. Then, we employed the *run10X* function of velocyto (v0.17.17) (La Manno *et al.* 2018) to subdivide the UMI counts into spliced and unspliced counts, generating a *loom* file containing the resulting matrices for each sample. Finally, we used the *build_adata_object* function of FIERCE to generate a new *anndata* object containing, in dedicated layers, the spliced and unspliced counts of all the merged samples. Using the same function, we added the cell annotations and embedding coordinates from the integrated Seurat object to the new object.

#### 2.3.2 FIERCE analysis

We applied the *perform_preprocessing* function of FIERCE to construct a neighborhood graph with 30 neighbors for each cell from the first 18 principal components. Then, we input the neighborhood graph to the *compute_future_states* function to compute the first-order moments of spliced and unspliced counts. For the signaling entropy computation, we used the built-in *high-confidence* murine PPI network of FIERCE, resulting in 12 857 genes retained from the original dataset. To construct the entropy-derived UMAP embedding, we computed 50 principal components from the observed local entropy scores and utilized all these components to construct a neighborhood graph with 100 neighbors for each cell. This graph was also used during the vector field construction to compute the velocity graph and the embedded velocities.

#### 2.3.3 scVelo analysis

The scVelo algorithm was applied as part of the FIERCE pipeline following the default procedure for the dynamical model (Bergen *et al.* 2020).

#### 2.3.4 Slingshot analysis

We first used the same neighborhood graph generated by the *perform_preprocessing* function of FIERCE to generate a set of cell clusters with the Leiden algorithm. Then, we provided such clusters to the Slingshot algorithm (v2.7.0) (Street *et al.* 2018) to generate a set of principal curves on the space defined by the first two principal components. To maintain unsupervised conditions, no root cluster was set. The root was automatically set by Slingshot in correspondence with the origin node shared by all the principal curves. Since, for each cell, a separate pseudotime score was computed for each curve onto which the cell was projected, we computed the cell-wise mean of such scores to obtain a single pseudotime value for each cell.

### 2.4 Hematopoiesis dataset

#### 2.4.1 Preprocessing

We downloaded the fastq files of the *in vitro* murine hematopoiesis dataset sequenced by Weinreb *et al.* (2020) with the LARRY protocol from the Sequence Read Archive (SRX7199569). Raw data comprised 32 libraries derived from cells sampled and sequenced using the inDrops (Klein *et al.* 2015) and Illumina technology after 2, 4, and 6 days of culture, resulting in 23 848 features profiled across a total of 123 942 cells. The reads were aligned to the mm10 mouse reference genome using STAR (v2.7.3) (Dobin *et al.* 2013), and the transcript count matrices were generated using the dropEst pipeline (v0.8.5) (Petukhov *et al.* 2018). We utilized the *run-dropest* function of velocyto (v0.17.17) (La Manno *et al.* 2018) to generate *loom* files with the transcript counts subdivided into spliced and unspliced. Finally, we used the *build_adata_object* function of FIERCE to merge the matrices of all libraries into a single *anndata* object for the subsequent analysis. The same function was also utilized to incorporate several cell annotations provided by the authors of the original study into the newly generated *anndata* object, including cell type labels, sampling time points, and clonal identities provided by the LARRY protocol. Using these identities, we filtered the dataset to retain only the cells successfully assigned to a specific clone, resulting in a total of 47 901 cells distributed as outlined in Table 3, available as supplementary data at *Bioinformatics* online. We then applied the FIERCE *perform_preprocessing* function to generate the neighborhood graph necessary for initiating the FIERCE pipeline. Data were log-normalized with a scale factor of 10 000, and 1230 highly variable genes were identified using the default procedure of the Scanpy package (Wolf *et al.* 2018). Subsequently, we scaled the data and performed regression on the total number of UMI counts per cell, the total number of detected genes per cell, and the fraction of counts of each cell falling on mitochondrial genes. Finally, we performed PCA generating, from the first 50 principal components, a neighborhood graph with 30 neighbors for each cell that was then used to compute the UMAP embedding.

#### 2.4.2 Differential gene expression and Gene Ontology enrichment analysis

To investigate the broad range of total signaling entropy scores observed among the multipotent progenitor cells, we performed differential gene expression analysis between high and low-entropy progenitors, followed by Gene Ontology enrichment analysis of the differentially expressed genes. Multipotent progenitors were first ranked according to their total signaling entropy scores. Cells in the upper quartile were defined as high-entropy progenitors, whereas cells in the lower quartile were defined as low-entropy progenitors. Differentially expressed genes between the two groups were identified using the *rank_genes_groups* function of the Scanpy package, with significance defined as a Benjamini–Hochberg adjusted *P*-value <.05. Genes upregulated in each group were then analyzed separately using the DAVID (Sherman *et al.* 2022) web application to identify enriched Gene Ontology Biological Process terms.

#### 2.4.3 FIERCE analysis

To start the FIERCE pipeline, we applied the *compute_future_states* function to quantify the first-order moments using the previously constructed neighborhood graph. During the signaling entropy computation step, we utilized the built-in *high-confidence* murine PPI network, retaining 14 428 genes from the original matrix. To construct the entropy-derived UMAP embedding, we computed 50 principal components from the observed local entropy scores and used all these components to construct a neighborhood graph with 30 neighbors for each cell. For computing the velocity graph and the embedded velocities, we used a different neighborhood graph with 100 neighbors to better capture the long-term entropy dynamics in this large dataset.

#### 2.4.4 scVelo analysis

The scVelo algorithm was applied as part of the FIERCE pipeline following the default procedure for the dynamical model (Bergen *et al.* 2020).

#### 2.4.5 Slingshot analysis

Slingshot analysis was performed as previously described for the MG development dataset.

#### 2.4.6 Monocle 3 analysis

We used Monocle 3 (Cao *et al.* 2019) to preprocess the raw gene expression data, perform PCA, and construct the UMAP embedding from 50 principal components. During preprocessing, we applied a batch correction procedure and performed regression on the total number of UMI counts per cell, the total number of detected genes per cell, and the fraction of counts of each cell falling on mitochondrial genes using the *batchelor* R package (v1.10.0) (Haghverdi *et al.* 2018). We applied the Monocle 3 default procedure to build the principal graph and compute the pseudotime scores of cells based on their projections on this graph. For this latter step, we utilized the *get_earliest_principal_node* function to manually set the cells that were sampled at culture Day 2 as the root of the genealogy (see https://cole-trapnell-lab.github.io/monocle3/docs/trajectories/ for details on the *get_earliest_principal_node* function).

#### 2.4.7 Inference of *ground-truth* and *predicted* cell fates

The LARRY protocol offers an invaluable opportunity to track the fate of cell clones across the sequence of states in a dynamic process throughout the entire experiment (Weinreb *et al.* 2020). Therefore, we decided to assess FIERCE’s ability to reconstruct reliable trajectories by comparing the known fates of cells with the fates inferred from their velocity of the entropy. First, to ensure a one-to-one comparison, we assigned a unique *ground-truth* fate to each cell based on its cell type label, sampling time point, and clonal identity information. We specifically selected cells from clones that could be unequivocally associated with a single differentiation lineage (i.e. those clones that transitioned from the multipotent progenitors’ basin to just one differentiated cell type during the experiment). We also included clones that remained within the multipotent basin throughout the experiment, without entering any differentiation lineage, as they likely represent multipotent cells undergoing self-renewal. Following this selection process, cells were assigned a *ground-truth* fate as follows: (i) cells from clones comprising solely multipotent progenitors were designated with the fate of multipotent progenitors and (ii) cells from clones containing both multipotent progenitors and one of the differentiated cell types were assigned the fate corresponding to that specific cell type. Next, we applied several filters to the resulting dataset of *single-fate* clones to ensure the retention of only those clones most likely to exhibit detectable transcriptional changes. Specifically, we retained clones containing cells from at least two sampling time points. Among these, we kept only clones with a cell type label distribution across the two or three time points compatible with a realistic transition from the multipotent state to one of the differentiated states. We excluded two types of clones: (i) those composed solely of differentiated cells in earlier time points and exclusively multipotent progenitors in later time points and (ii) clones consisting solely of multipotent progenitors in the first and third time points and exclusively differentiated cells in the second time point. The rationale behind this latter filter was to exclude clones that, due to uneven sampling, exhibit an implausible distribution of cell type labels across subsequent time points. After applying these filters, we removed all cells assigned to the erythroid, eosinophil, plasmacytoid dendritic, or migratory dendritic fate, as these fates were assigned to <100 cells each, making it unlikely for them to be identified as separate lineages. The *ground-truth* fate of all 21 726 cells that remained after applying these filters is listed in Table 4, available as supplementary data at *Bioinformatics* online. All subsequent analyses were conducted using this dataset.

For comparing the *ground-truth* fates with those inferred from the velocity of the entropy of each cell, we utilized CellRank 2, an algorithm specifically designed to reconstruct the long-term fates of cells using various dynamic information, including velocity metrics (Lange *et al.* 2022, Weiler *et al.* 2024). Specifically, we first used the *VelocityKernel* function of CellRank 2 to calculate a cell-cell transition matrix utilizing the velocity of the entropy and then applied the GPCCA estimator (Reuter *et al.* 2019) to identify the terminal states of the dynamic process and to compute the confidence level for assigning each cell to these terminal states. The terminal states were named according to the *ground-truth* fates listed in Table 4, available as supplementary data at *Bioinformatics* online, and the state showing the highest confidence was selected as the predicted fate for each cell. To assess the prediction accuracy of the velocity of the entropy, we applied the CellRank 2 analysis also using classic RNA velocity and the pseudotime score of Slingshot as dynamic measures. The cell-cell transition matrix was computed using the *VelocityKernel* and the *PseudotimeKernel* functions of CellRank 2 for RNA velocity and pseudotime score, respectively.

#### 2.4.8 Simulated trajectories

We used the dyngen simulation engine (v1.0.5) (Cannoodt *et al.* 2021) to simulate a total of 10 medium-sized scRNA-seq datasets of 5000 cells each. Each dataset was designed to represent a simple bifurcating trajectory with a single branching point; each branch of such trajectory is divided into two sections, for a total of six sections. The cells of each section are characterized by the progressive upregulation of specific gene modules comprising 10 regulatory genes (transcription factors) each. We instructed dyngen to simulate, for each dataset, the expression counts (both spliced and unspliced) of the transcription factors within the gene modules plus 10 000 target genes, organized in a gene regulatory network. We then applied FIERCE, scVelo, Monocle 3, and Slingshot to each simulated dataset using the same procedures described above for the hematopoiesis dataset, with the following exceptions: (i) during the scaling procedure of the expression matrices, no regressions were performed, and the following PCA was performed on all genes rather than just on the highly variable ones, (ii) during the FIERCE analysis, the simulated gene regulatory network of each dataset was used as the PPI network for signaling entropy computation, and (iii) at the end of the FIERCE analysis, a neighborhood graph with 30 neighbors, in place of 100, was constructed from the entropy values to generate the vector field of the velocity of the entropy.

To assess the accuracy of each method in reconstructing the known trajectories, we devised an accuracy score that measures how well the reconstructed trajectories recapitulate the known expression trends of key transcription factors. Specifically, for each simulated dataset, we first selected, for each section of the trajectory, the transcription factors belonging to gene modules defined as upregulated by the dyngen model. Then, given the cells included in the selected trajectory section, we computed the mean expression variation of each transcription factor *g* across the cell-cell transitions (*origin–destination*) predicted by each method:


(9) $$\mathrm{mean}{\Delta \;\text{exp}\;}_{g}=\frac{\sum_{1}^{n_{c}}\;{\text{exp}}_{g,\mathrm{destination}}-{\text{exp}}_{g,\mathrm{origin}}}{n_{c}}$$

where $n_{c}$ is the total number of cells included in each section and $\;{\text{exp}}_{g}$ are the normalized counts. The cell-cell transitions predicted by each method were defined by choosing, for each cell of the selected section taken as *origin*, the *destination* cell toward which it had the maximum probability to transition according to the cell-cell transition probability matrix defined by each method; in case of ties, the mean expression of the *destination* cells was used. To account for the known trajectory direction, we included in the above formula only the cell-cell transitions where the *destination* cell belonged either to the same trajectory section of the *origin* cell, or to the following section according to the known trajectory order. The transition probability matrices of FIERCE and scVelo were computed during their respective pipelines; for the two pseudotime-based methods (i.e. Monocle 3 and Slingshot) we used the *compute_transition_matrix* function of CellRank 2 to compute the transition matrices from the pseudotime values. The final accuracy score AS was computed by computing, for each trajectory, the proportion of transcription factors *g* with a positive mean expression variation $\mathrm{mean}{\Delta \;\text{exp}\;}_{g}$:


(10) $$AS=\frac{\sum \mathrm{mean}{\Delta \;\text{exp}\;}_{g}>0}{\sum \mathrm{mean}{\Delta \;\text{exp}\;}_{g}}$$

This accuracy score quantifies how many transcription factors, expected to increase in expression along specific sections of the trajectory, are indeed upregulated when cells are ordered according to the trajectory constructed by each method.

To assess the performance of FIERCE, scVelo, Monocle 3, and Slingshot under reduced dataset sizes, we progressively downsampled 5 of the 10 simulated datasets by retaining 2500, 1000, and 500 cells. Cells were selected by stratified random sampling to preserve the relative abundance of each section of the simulated trajectory. Each of the four methods was then applied to each downsampled dataset using the same procedure described above, and the accuracy score was computed for each method-subsample combination.

## 3 Results

### 3.1 FIERCE workflow

**Figure 1:**
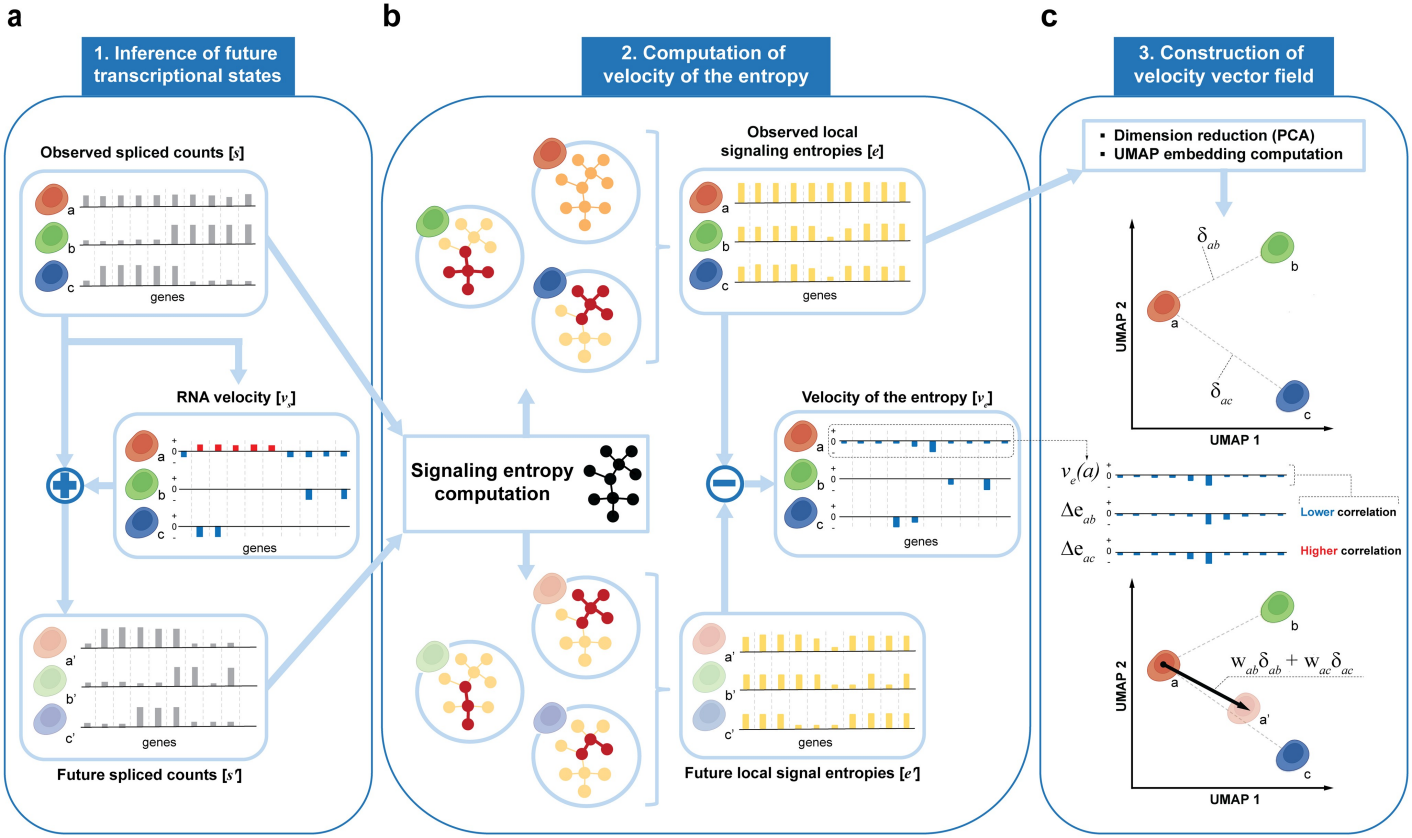
Schematic of FIERCE workflow. (a) In the first step, the observed spliced transcript counts [*s*] are added to the corresponding RNA velocities [*v_s_*], defined as the rate of change of spliced counts over time, to predict the future spliced transcript counts [*s*′] of all genes in all cells. (b) In the second step, the local signaling entropy scores of all genes in all cells are computed from both the observed [*s*] and the future spliced counts [*s*′], based on their distribution over the edges of a cell-shared genome-wide PPI network. The observed local signaling entropy scores [*e*] are then subtracted from the corresponding future scores [*e*′] to obtain the velocity of the entropy [*v_e_*] of each gene in each cell, which represents the rate of change of its local signaling entropy over time. (c) In the final step, a UMAP embedding is constructed directly from the observed local signaling entropy scores to obtain a structural representation of the differentiation potency landscape of the system. Then, for each given cell (e.g. cell a), a series of cosine correlations is computed between the vector containing the velocity of the entropy [*ve*] of all its genes and each vector containing the differences [Δ*e*] between its local entropy scores and the scores of all its neighbors on the entropy space (e.g. cells b and c). Finally, the future displacement of cell a in the embedding (i.e. a′) is inferred as a weighted sum of its distances [δ] from all its neighbors, where the weights [*w*] are a function of the previously computed cosine correlations. The figure contains elements adapted from BioRender.com.

FIERCE is a computational pipeline that reconstructs both the structure and the future dynamics of the differentiation potency landscapes of cell populations from scRNA-seq data. It has been designed according to a fully *bottom-up* strategy based on the following rationale: if we assume that the differentiation potency of cells is efficiently approximated by their transcriptional entropy, it is possible to reconstruct dynamic trajectories directly on the potency landscape of cell populations through (i) computing the rate of change of the transcriptional entropy of cells over time and (ii) predicting the future movements of cells in the entropy space based on this rate of change. We implemented this concept into a three-step process, as illustrated in Fig. 1 and briefly explained below. For a more detailed description, please refer to Section 2.

The first step of the FIERCE pipeline involves inferring the future transcriptional states of all cells based on the RNA velocity of their genes, which corresponds to the rate of change of the spliced transcript counts of those genes over time (La Manno *et al.* 2018, Bergen *et al.* 2020) (Fig. 1a). Specifically, the observed spliced counts [s] are summed to the corresponding RNA velocities [$v_{s}$] to predict the future spliced transcript counts [s′] of all genes in all cells. The predicted future spliced counts [s′] represent the *dynamic* information contained within the dataset, which can be used to infer the future behavior of any quantity that can be computed from transcriptional data, including transcriptional entropy.

In the second step, FIERCE infers the future dynamics of the transcriptional entropies of all cells (Fig. 1b). To define transcriptional entropies, we adopted the signaling entropy score, which measures the distribution of the gene expression signal of cells across a shared genome-wide protein-protein interaction (PPI) network containing all known interactions between gene products (Banerji *et al.* 2013, Teschendorff and Enver 2017). The total signaling entropy score of a cell is directly proportional to the level of dispersion of the expression signal across the network. Based on the assumption that the expression signal of high-potency cells is broadly distributed across a wide variety of pathways within the PPI network, these cells are expected to exhibit a high signaling entropy score. Conversely, low-potency cells are expected to present a low signaling entropy score because their expression signal is presumed to be predominantly concentrated on a few specific pathways within the PPI network (MacArthur and Lemischka 2013, Teschendorff and Enver 2017). For each cell, a local signaling entropy score can also be computed for each gene within the PPI network based on the local dispersion of the expression signal (Teschendorff and Enver 2017). FIERCE calculates the local signaling entropy scores of all genes in all cells first from the observed spliced transcript counts and then from the future spliced counts (computed in the first step), to obtain the observed [e] and the future [e′] local signaling entropy scores, respectively. These scores represent the observed and future differentiation potency profiles of the cell population. Thus, by subtracting the observed from the future profiles, we can ultimately estimate the rate of change of the potency of cells during a time unit, represented by the rate of change of the local entropy scores of their genes (Fig. 1b). We refer to this rate as the *velocity of the entropy* [$v_{e}$]. This metric integrates the static information provided by signaling entropy with the dynamic information provided by RNA velocity, and thus constitutes the key feature used by FIERCE to reconstruct the dynamics of cells in the potency space.

In the final step, FIERCE first constructs a 2D representation of the potency landscape of the cell population by computing a uniform manifold approximation and projection (UMAP) embedding directly from the observed local signaling entropy scores of all cells (Fig. 1c). Then, it constructs the vector field of the velocity of the entropy on such embedding by employing the same principle used to construct the RNA velocity vector field in the gene expression space (La Manno *et al.* 2018, Bergen *et al.* 2020). The future *displacement* of each cell on the UMAP embedding (i.e. the distance between its current and future position) is predicted as a weighted sum of its distances [δ] from all its neighbors on this embedding. For any given cell, each weight is proportional to the correlation between the velocity of entropy [$v_{e}$] of its genes and the difference [Δe] between its local entropy scores and the scores of each neighbor in the entropy space (Fig. 1c). The rationale behind this procedure is that, as the dynamic process unfolds, cells tend to move toward points on the signaling entropy landscape that show the strongest correlation with their predicted entropy change. Analogous to its counterpart constructed from classic RNA velocity, the vector field of the velocity of the entropy depicts both the structure and the predicted future dynamics of the cell population within a single image. The fundamental difference between the two vector fields is that the vector field of the velocity of the entropy is constructed directly on a representation of the differentiation potency landscape, aiming to provide the most accurate and interpretable depiction of the underlying dynamic process.

### 3.2 Reconstruction of murine pancreas endocrinogenesis

**Figure 2:**
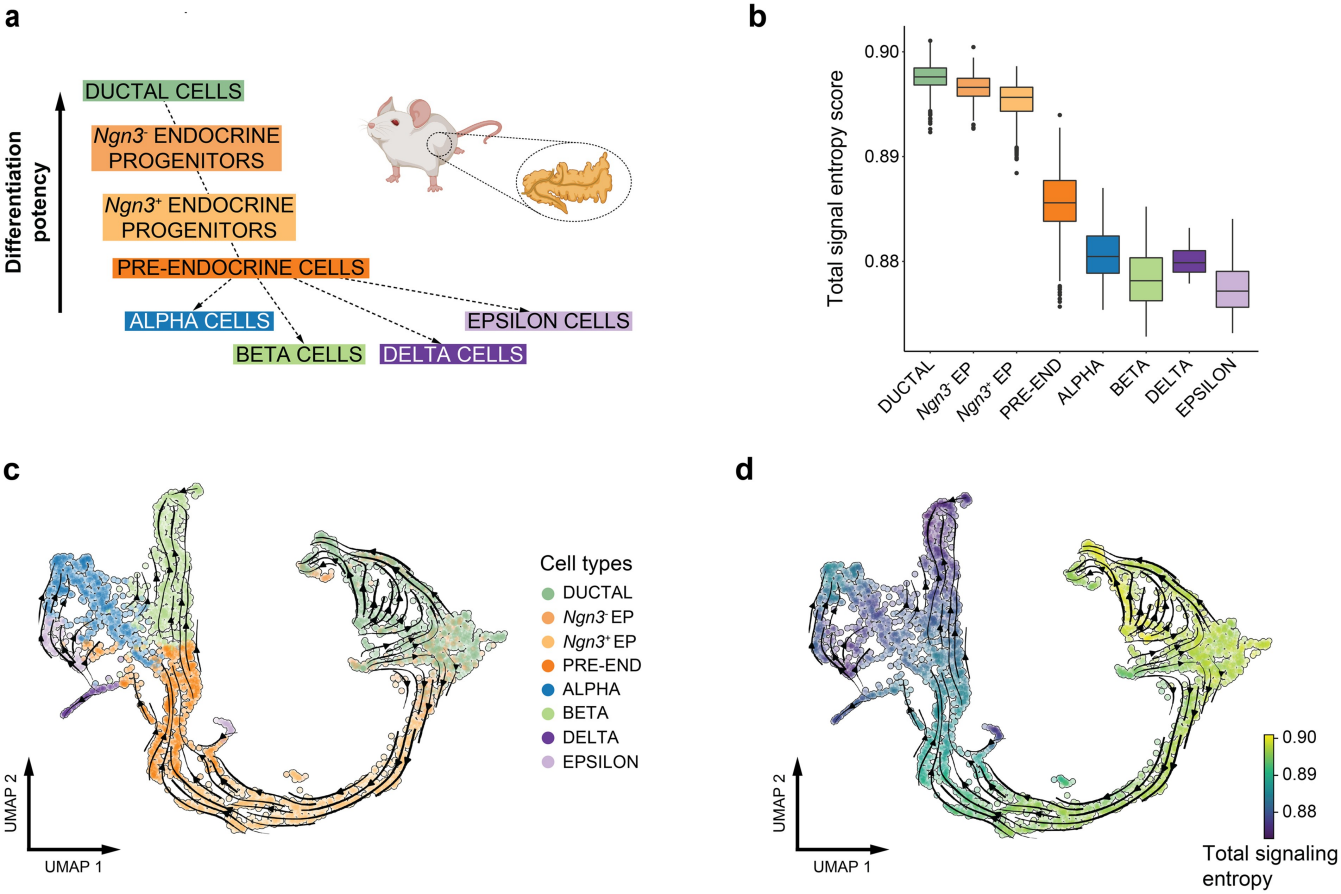
Application of FIERCE to scRNA-seq data from murine pancreas endocrinogenesis. (a) Expected genealogy of the differentiation process during pancreas endocrinogenesis. (b) Distribution of the total signaling entropy scores across the cell types. (c and d) Vector field of the velocity of the entropy constructed on the entropy-derived UMAP embedding; cells are colored according to (c) cell type labels and (d) total signaling entropy scores. DUCTAL, ductal cells; Ngn3- EP, Ngn3- endocrine progenitors; Ngn3+ EP, Ngn3+ endocrine progenitors; PRE-END, preendocrine cells; ALPHA: alpha cells, BETA: beta cells, DELTA, delta cells; and EPSILON, epsilon cells. The figure contains elements adapted from BioRender.com.

We tested FIERCE on three scRNA-seq datasets to reconstruct three murine developmental processes characterized by different dynamics and complexity. The first dataset, comprising 3696 cells, describes the differentiation of four endocrine cell types (i.e. alpha, beta, delta, and epsilon cells) from their ductal progenitors during pancreas development in mouse embryos (Fig. 2a; Table 1, available as supplementary data at *Bioinformatics* online) (Bastidas-Ponce *et al.* 2019). The distribution of the total signaling entropy scores computed by FIERCE across the cell types is characterized by an expected decreasing trend. Ductal progenitor cells exhibit the highest scores, fully differentiated endocrine cells show the lowest scores, and transient endocrine progenitors and preendocrine cells display intermediate scores (Fig. 2b). Remarkably, endocrine progenitors expressing the *Ngn3* gene exhibit lower scores compared to endocrine progenitors that do not express this gene, consistent with the idea that the activation of *Ngn3* determines the commitment of this subpopulation to the endocrine fate (Bastidas-Ponce *et al.* 2019). This pattern is further confirmed by grouping the cells into potency states (i.e. clusters of cells with specific distributions of total signaling entropy scores; see Section 2; Fig. 1a, available as supplementary data at *Bioinformatics* online). These results confirm the efficacy of signaling entropy as a measure of the differentiation potency of cells, thus highlighting its suitability for FIERCE’s *bottom-up* approach. The UMAP embedding generated by FIERCE from the local signaling entropy scores, along with the vector field of the velocity of the entropy, predicts the dynamics of cells on the differentiation potency landscape (Fig. 2c and d). The arrows converge into a prominent stream that originates at the junction between ductal cells and endocrine progenitors, passes through the intermediate cell states, and spreads across the terminally differentiated endocrine cells, completely aligning with the expected genealogy (Fig. 2a). This stream is accompanied by a gradual decrease in the total signaling entropy scores of cells (Fig. 2d; Fig. 1b, available as supplementary data at *Bioinformatics* online).

### 3.3 Reconstruction of murine mammary gland development

**Figure 3:**
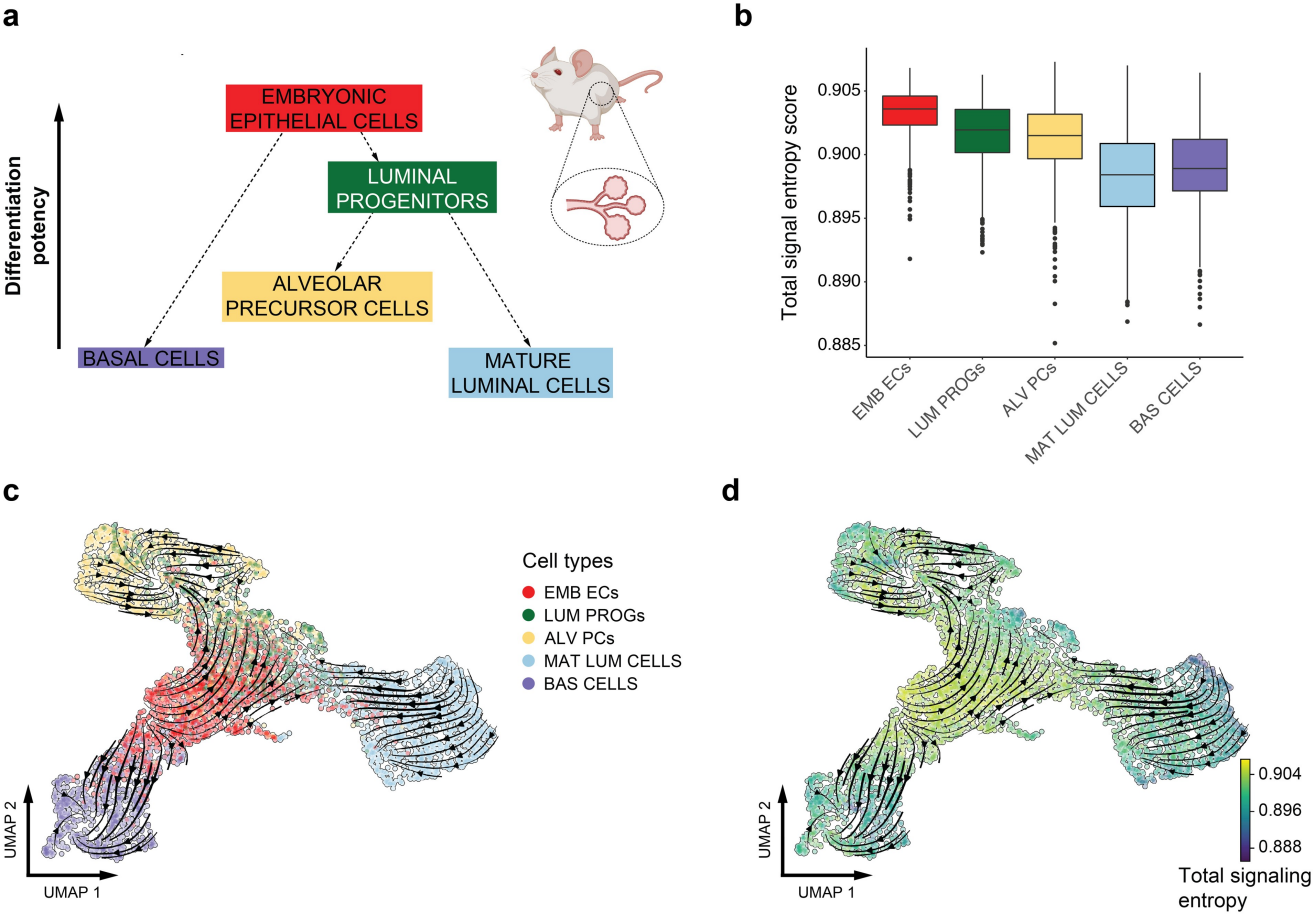
Application of FIERCE to scRNA-seq data from mouse mammary gland development. (a) Expected genealogy of the differentiation process during mouse mammary gland development. (b) Distribution of the total signaling entropy scores across the cell types. (c and d) Velocity of the entropy vector field constructed on the entropy-derived UMAP embedding; cells are colored according to (c) cell type labels and (d) total signaling entropy scores. EMB ECS, embryonic epithelial cells; LUM PROGS, luminal progenitors; ALV PCS, alveolar precursor cells; MAT LUM CELLS, mature luminal cells; BAS CELLS, basal cells. The figure contains elements adapted from BioRender.com.

We then applied FIERCE to the analysis of the MG development in mouse. This complex developmental system involves multiple branching events: initially, embryonic MG epithelial cells give rise to basal cells and luminal progenitors, followed by the latter giving rise to mature luminal cells and alveolar precursor cells through a second bifurcation (Fig. 3a). To reconstruct this genealogy, we analyzed a subset of 5168 cells from the scRNA-seq dataset described in Giraddi *et al.* (2018) (Table 2, available as supplementary data at *Bioinformatics* online). The distribution of the total signaling entropy scores computed by FIERCE across cell types is consistent with expectations: embryonic MG epithelial cells exhibit the highest scores, basal and mature luminal cells display the lowest scores, and luminal progenitors fall in between (Fig. 3b). Alveolar precursor cells present only slightly lower levels of signaling entropy compared to luminal progenitors, suggesting that they might still maintain the potential to further differentiate into other cell types. This is compatible with the fact that these cells become fully differentiated only after pregnancy, when they form the alveoli and start to produce milk (Visvader and Lindeman 2011). Interestingly, basal cells display an average entropy score higher than mature luminal cells, which represent the most differentiated MG epithelial cell population. This suggests that basal cells may still have some differentiation potency, in line with evidence that they contain a subpopulation endowed with some stem properties, at least upon transplantation (Van Keymeulen *et al.* 2011). This pattern is confirmed by the distribution of cell types across the three potency states identified by FIERCE (Fig. 2a, available as supplementary data at *Bioinformatics* online).

The streamplot generated by FIERCE accurately reconstructs both the structure and dynamics of this genealogy (Fig. 3c). Two distinct streams of arrows originate from the central core of embryonic epithelial cells and then diverge. One stream is directed toward the basal cells, while the other points in the opposite direction toward the luminal progenitors. Here, the arrows diverge again, with one set pointing toward the mature luminal cells and the other toward the alveolar precursors. At each endpoint of this tripartite structure, the arrows form vortex-like patterns, suggesting that cells have reached the stable state of their respective lineages. The distribution of the total signaling entropy scores on the streamplot aligns with the directions of the arrows in the vector field (Fig. 3d; Fig. 2b, available as supplementary data at *Bioinformatics* online), with a central entropy peak precisely located at the core of embryonic epithelial cells and three entropy sinks positioned at the three terminal states. The decreasing slopes of signaling entropy that connect the central peak to the three sinks correspond to the most prominent streams of arrows. These results demonstrate the effectiveness of the FIERCE *bottom-up* approach in reconstructing complex branching developmental processes without any prior specification of either the genealogy’s origin or the expected location of branching events.

**Figure 4:**
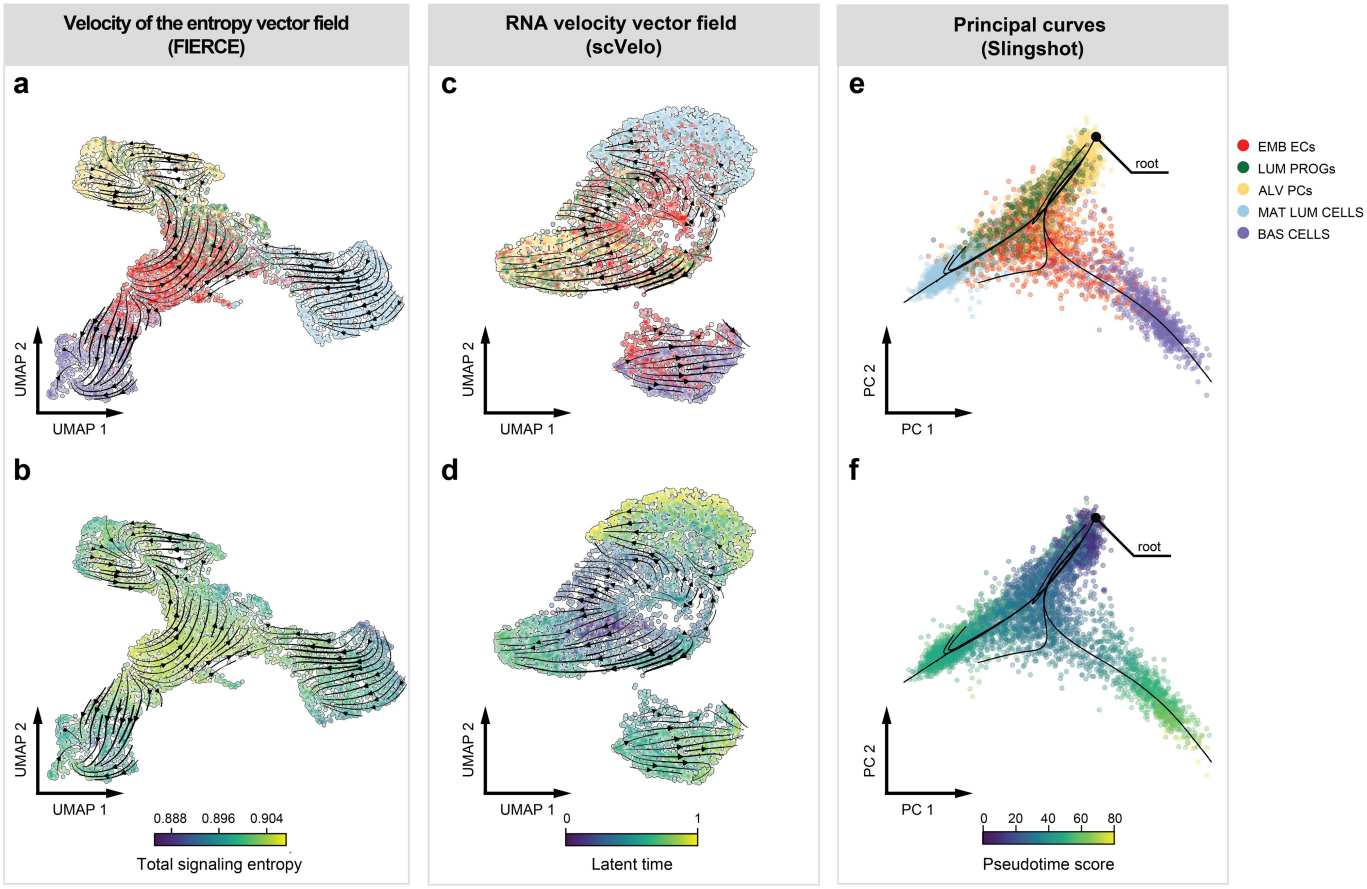
Comparison of the trajectories reconstructed by FIERCE, scVelo, and Slingshot using the scRNA-seq data of the mammary gland development dataset. (a and b) The vector field of the velocity of the entropy reconstructed by FIERCE; cells are colored according to (a) the cell type labels and (b) to the total signaling entropy scores. (c and d) The vector field of RNA velocity is obtained using scVelo; cells are colored according to (c) cell type labels and (d)latent time. (e and f) The principal curves constructed by Slingshot; cells are colored according to (e) cell type labels and (f) pseudotime scores. Abbreviations: EMB ECS: embryonic epithelial cells; LUM PROGS, luminal progenitors; ALV PCS, alveolar precursor cells; MAT LUM CELLS, mature luminal cells; BAS CELLS, basal cells.

We compared the vector field of the velocity of the entropy obtained by FIERCE (Fig. 4a and b) with the vector field of RNA velocity constructed by scVelo (Bergen *et al.* 2020) (Fig. 4c and d) and with the principal curves constructed by Slingshot (Street *et al.* 2018), one of the most popular and top-performing (Saelens *et al.* 2019) trajectory reconstruction algorithms based on the classic *top-down* approach (Fig. 4e and f). To maintain unsupervised conditions, we ran Slingshot without specifying the root, and let the algorithm estimate its position. The vector field of RNA velocity exhibits no interpretable pattern in the directions of the arrow streams, most likely because of the split structure of the underlying UMAP embedding derived from gene expression (Fig. 4c). Indeed, most embryonic epithelial cells are mixed with the cells of the luminal branch, and basal cells are in a separate cluster disconnected from the main genealogy. This ambiguous configuration is further emphasized by the distribution of the latent time scores, which represent the progression of cells along the RNA velocity dynamical model (Bergen *et al.* 2020) (Fig. 4d). Indeed, the latent time score is expected to be inversely proportional to the potency of cells, but in this case, in some areas of the embedding, cells with different latent times appear intermingled with each other. The principal curves constructed by Slingshot do not correctly recapitulate the expected branching structure: the root is erroneously located within the alveolar precursors, from which several trajectories depart terminating either into the other branches or back into the alveolar precursors themselves (Fig. 4e). Predictably, the pseudotime scores computed on such curves do not reliably reflect the differentiation stages of cells (Fig. 4f).

### 3.4 Reconstruction of *in vitro* murine hematopoiesis

**Figure 5:**
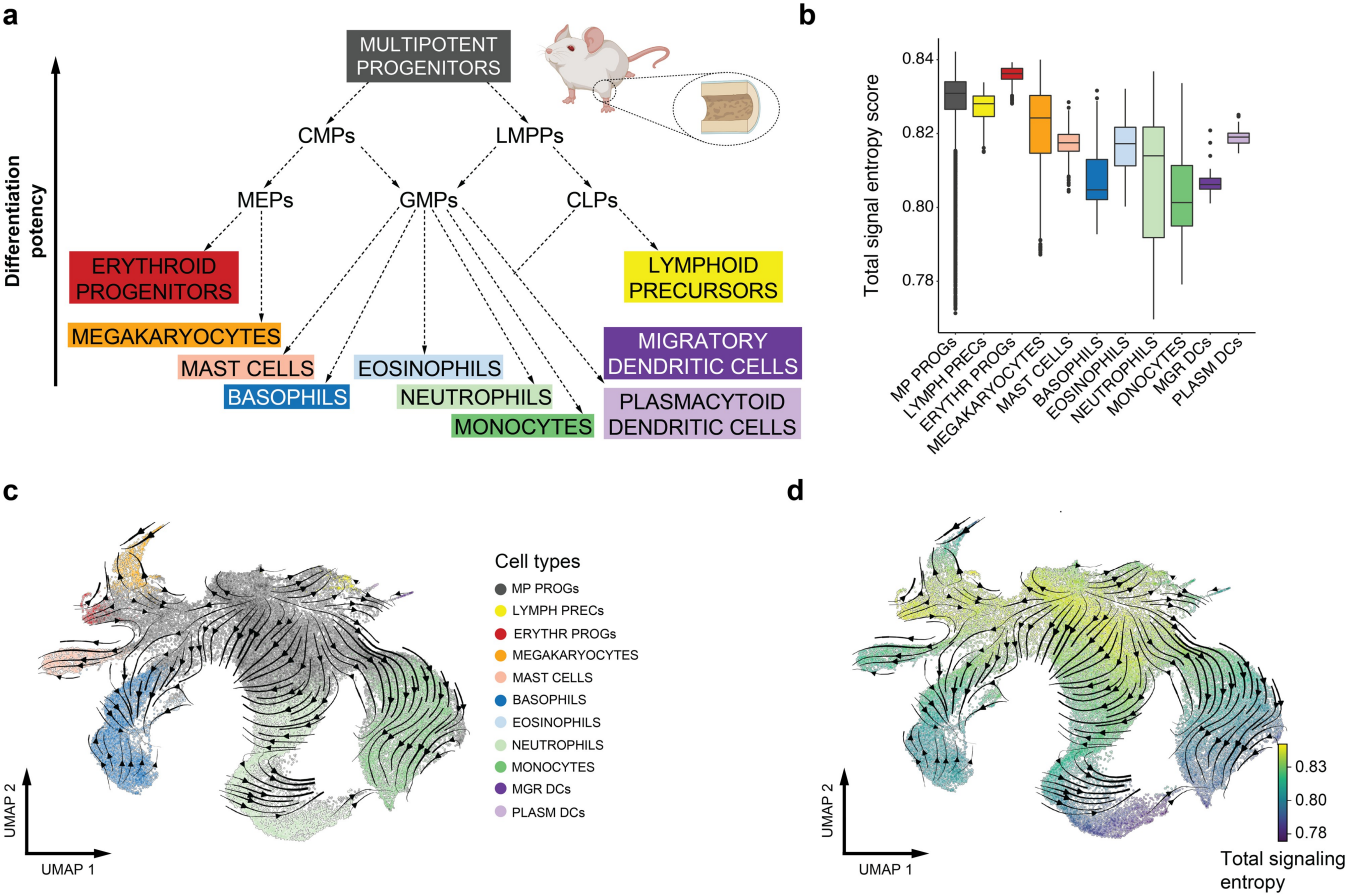
Application of FIERCE to scRNA-seq data from the *in vitro* murine hematopoiesis. (a) Expected genealogy of the *in vitro* murine hematopoiesis differentiation process (CMPs, common myeloid progenitors; LMPPs, lymphoid-primed multipotent progenitors; MEPs, megakaryocyte-erythrocyte progenitors; GMPs, granulocyte-monocyte progenitors; CLPs, common lymphoid progenitors). (b) Distribution of the total signaling entropy scores across the cell types. (c and d) Velocity of the entropy vector field constructed on the entropy-derived UMAP embedding; cells are colored according to (c) cell type labels and (d) total signaling entropy scores. MP PROGs, multipotent progenitors; LYMPH PRECs, lymphoid precursors; ERYTHR PROGs, erythroid progenitors; MGR DCs, migratory dendritic cells; PLASM DCs, plasmacytoid dendritic cells. The figure contains elements adapted from BioRender.com.

Finally, we tested FIERCE on a complex dynamic process where several distinct differentiation paths originate from a single multipotent population. Specifically, we examined the *in vitro* maturation of blood cells from hematopoietic stem and progenitor cells derived from murine bone marrow using the scRNA-seq data presented by Weinreb *et al.* (2020). This dataset served as a demonstration of a novel hybrid experimental protocol called LARRY (LineAge and RNA RecoverY), which combines scRNA-seq with lineage tracing to reconstruct the transcriptional evolution of cell clones across subsequent generations. The LARRY protocol enables the unique tagging of individual cell clones, allowing to track their trajectories across various cell states throughout the entire duration of the experiment (Weinreb *et al.* 2020). Thus, this dataset offers the unique opportunity to reconstruct dynamic trajectories while having access to experimentally determined *ground-truth* cell fates. Specifically, we analyzed the single-cell transcriptional profiles of a population of murine hematopoietic stem and progenitor cells that were barcoded using the LARRY protocol and subsequently cultured *in vitro* for 6 days. A total of 1 23 942 cells were sampled and sequenced at 2-day intervals (i.e. Days 2, 4, and 6). We considered only the 47 901 cells that could be successfully assigned to a specific clone. This subset comprises multipotent undifferentiated progenitors, lymphoid precursors, erythroid progenitors, and seven distinct blood and immune cell types (Table 3, available as supplementary data at *Bioinformatics* online). Figure 5a shows a schematic representation of the expected genealogy of this differentiation system, with multipotent progenitors gradually differentiating into oligopotent and unipotent progenitors that are committed to specific fates (Cheng *et al.* 2020). The distribution of the total signaling entropy scores quantified by FIERCE across cell types reveals that, as expected, multipotent progenitors exhibit very high scores (Fig. 5b). However, within this population, score values vary widely, with numerous outliers exhibiting low values. To further investigate this heterogeneity, we compared high- and low-entropy multipotent progenitors by differential gene expression analysis followed by Gene Ontology enrichment analysis. Genes upregulated in high-entropy progenitors were enriched for Gene Ontology terms associated with cell proliferation and RNA processing, whereas genes upregulated in low-entropy progenitors were enriched for terms associated with immune response and cell migration (Fig. 3, available as supplementary data at *Bioinformatics* online). These results support the interpretation that the broad entropy distribution observed among multipotent progenitors reflects a gradual transition from a high potency, relatively undifferentiated state toward lower potency lineage-committed states. Lymphoid precursors and erythroid progenitors also exhibit high scores, as expected given that they are still endowed with some differentiation potency, although limited to the lymphoid and erythroid lineages, respectively (Tusi *et al.* 2018). Conversely, the distributions of most other cell types are centered around lower values, consistent with their more advanced differentiation stage. Notably, megakaryocytes are exceptions, as they exhibit levels of total signaling entropy similar to those of multipotent progenitors. We hypothesize that these cells might still be in an early stage of differentiation (i.e. that they might have just branched from the common progenitors they share with erythroid precursors (Cheng *et al.* 2020)). This is supported by the short time these samples spent in the differentiation medium (at most 6 days), which might have been insufficient for a complete differentiation. These observations are reflected in the distribution of cell types across the 5 potency states identified by FIERCE (Fig. 4a, available as supplementary data at *Bioinformatics* online).

The vector field of the velocity of the entropy, constructed by FIERCE on the UMAP embedding from the local signaling entropy scores, shows that multipotent progenitors form a uniform central mass, with several branches extending outward, each culminating in a different cell type (Fig. 5c). Each branch corresponds to a distinct stream of arrows originating from the entropy peak of the central multipotent mass and then following its own trajectory along the corresponding descending entropy gradient (Fig. 5d; Fig. 4b, available as supplementary data at *Bioinformatics* online). This pattern resembles the time spent by cells in the culture medium: cells sampled on Day 2 are concentrated near the entropy peak, cells sampled on Day 4 are distributed along the branches, and cells sampled on Day 6 progressively concentrate in the entropy sinks at the ends of the various branches (Fig. 4c, available as supplementary data at *Bioinformatics* online). These results are compatible with the expected gradual differentiation of distinct cell lineages (Fig. 5a). Interestingly, the embedding appears subdivided into two sections, with megakaryocytes, erythroid progenitors, mast cells, basophils, and eosinophils forming a separate branch from neutrophils, monocytes, dendritic cells, and lymphoid precursors. This bipartite structure is consistent with a recent reconstruction of the early mouse hematopoietic hierarchy (Tusi *et al.* 2018). Notably, both branches also include a portion of multipotent progenitors, likely representing a continuum of committed differentiation intermediates leading to specific cell fates. Erythroid progenitors, megakaryocytes, and lymphoid precursors exhibit less pronounced entropy gradients compared to other cell types (Fig. 5c and d). Moreover, in the region of the erythroid progenitors, the arrows predominantly move toward neighboring megakaryocytes and multipotent progenitors rather than toward the periphery of the embedding (Fig. 5c). Considering that erythrocytes and megakaryocytes share the same intermediate progenitors, these observations support the hypothesis that the differentiation of these cells may still be in a very early stage, potentially hampering their distinct separation from the multipotent basin.

**Figure 6:**
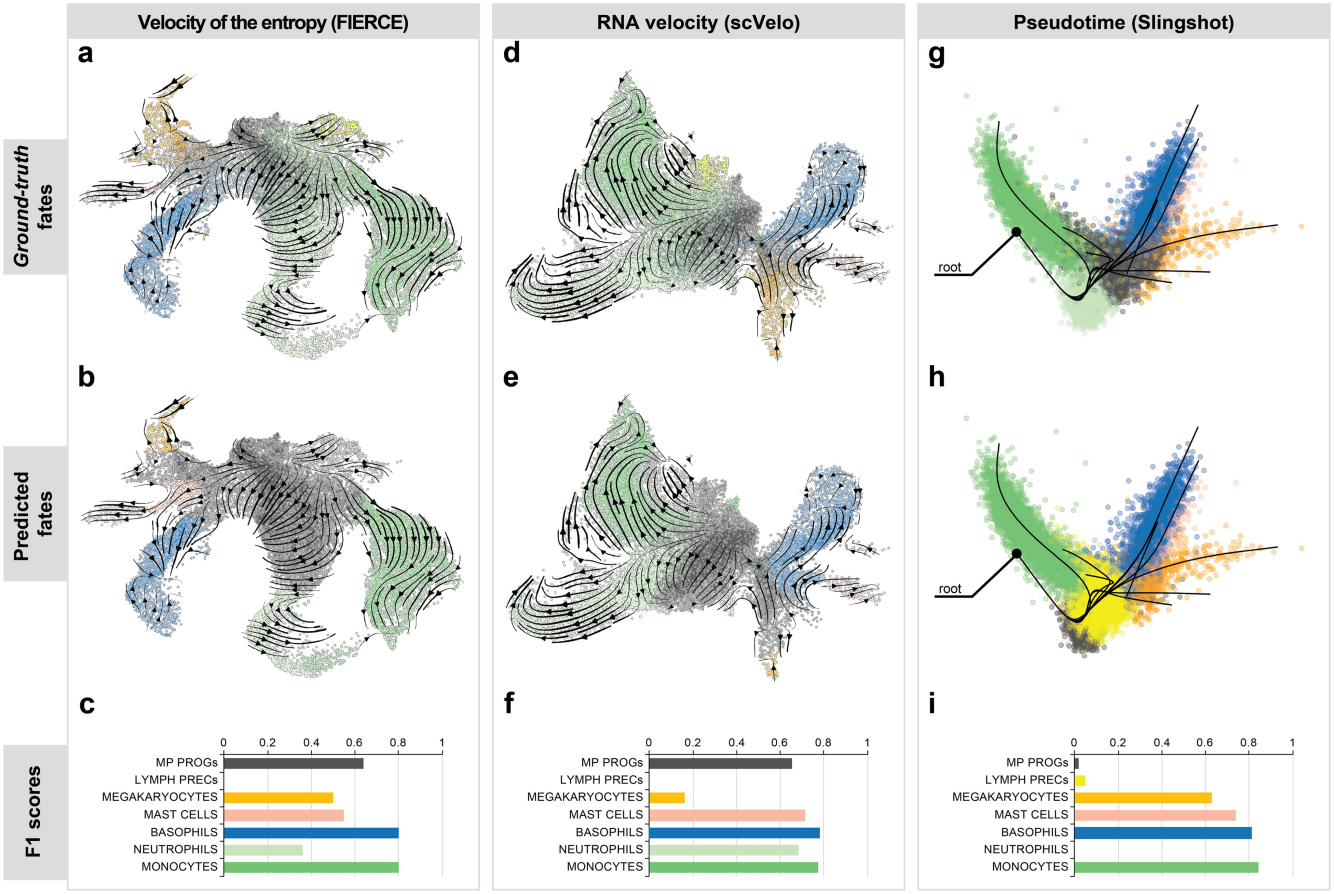
Comparison between the ground-truth cell fates of the *in vitro* murine hematopoiesis dataset and the fates predicted by the CellRank 2 algorithm based on different metrics. (a and b) The velocity of the entropy vector field constructed by FIERCE and projected on the entropy-derived UMAP embedding; cells are colored according to (a) the ground-truth fates and (b) the predicted fates. (c) F1 scores of the predictions for each cell fate based on the velocity of the entropy computed by FIERCE. (d and e) The RNA velocity vector field constructed by scVelo; cells are colored according to (d) the ground-truth fates and (e) the predicted fates. (f) F1 scores of the predictions for each cell fate based on the RNA velocity computed by scVelo. (g and h) The principal curves constructed by Slingshot; cells are colored according to (g) the ground-truth fates and (h) the predicted fates. (i) F1 scores of the predictions for each cell fate based on the pseudotime score computed by Slingshot. Cells are color-coded as in Fig. 5c.

To quantitatively evaluate the accuracy of the trajectories that were reconstructed by FIERCE, we first used the dynamic information of the velocity of the entropy to predict the long-term differentiation fate of each cell. Then, we compared this prediction with the respective *ground-truth* fate evinced from the clonal information provided by the LARRY protocol. To predict cell fates from the reconstructed trajectories, we employed a separate algorithm (i.e. CellRank 2 (Lange *et al.* 2022, Weiler *et al.* 2024)) which assigns cells to terminal states using any dynamic measure that defines their progression along a differentiation process. In our specific case, we used the velocity of the entropy computed by FIERCE as a dynamic measure (Fig. 5c). For comparison, we also used the classic RNA velocity (computed by scVelo; Fig. 5, available as supplementary data at *Bioinformatics* online) and the pseudotime score of Slingshot (run in unsupervised mode without manually setting the root; Fig. 6, available as supplementary data at *Bioinformatics* online). To assign the *ground-truth* fates, we combined clonal identities resulting from the LARRY protocol and cell type annotations (see Section 2 for details). For all methods, the analysis was restricted to the 21 726 cells exclusively assigned to a single *ground-truth* fate with at least 100 cells. This selection removed the erythroid, eosinophil, plasmacytoid dendritic, and migratory dendritic fates that comprised <100 cells each (Fig. 6; Figs 5–7 and Table 4, available as supplementary data at *Bioinformatics* online).

For each dynamic measure, we visualized the distribution of *ground-truth* and *predicted* fates on the corresponding embedding, and calculated their concordance using the F_1_ score, which combines precision and recall (Taha and Hanbury 2015) (Fig. 6; Table 5, available as supplementary data at *Bioinformatics* online). Overall, the velocity of the entropy and RNA velocity exhibit similar predictive accuracy, with mean F_1_ scores of 0.52 and 0.54, respectively. Both dynamic measures accurately reconstructed the lineages of multipotent progenitors, basophils, and monocytes. However, they both failed to identify lymphoid precursors as a separate lineage, erroneously assigning cells destined for this fate to the multipotent progenitor fate (Fig. 6a–f). The pseudotime score of Slingshot exhibited the poorest performance; this measure achieves a mean F_1_ score of 0.44, fails to identify the neutrophil fate, and assigns most cells destined for the multipotent progenitor fate to the lymphoid precursor fate (Fig. 6g–i). Indeed, Slingshot pseudotime score is the only measure that identifies the lymphoid precursor fate, but this is achieved at a very low F_1_ score due to the low precision (Table 5, available as supplementary data at *Bioinformatics* online). The poor performance of all three measures in identifying this specific fate suggests that lymphoid precursors are still too immature to be distinguished from the nearby multipotent progenitors and recognized as a distinct lineage. Nevertheless, it is worth noting that the velocity of the entropy vector field succeeds in discerning the nascent branch of lymphoid precursors through a distinct stream of arrows (Figs 5c and 6a), while the RNA velocity vector field shows no arrows pointing to this subpopulation (Fig. 6d; Fig. 5, available as supplementary data at *Bioinformatics* online). Moreover, while RNA velocity achieved higher accuracy in predicting the fates of mast cells and neutrophils, the velocity of the entropy performed better in predicting the fate of megakaryocytes (Fig. 6c and f; Table 5, available as supplementary data at *Bioinformatics* online). Remarkably, the RNA velocity vector field depicts ambiguous trajectories in this section of the embedding, with arrows spawning within the megakaryocytes and proceeding toward basophils and mast cells through the multipotent progenitors (Fig. 6d; Fig. 5, available as supplementary data at *Bioinformatics* online). Conversely, the vector field of the velocity of the entropy delineates a clearer scenario, with basophils and mast cells defined by their own differentiation trajectories that originate in the multipotent progenitor basin (Figs 5c and 6a). Regarding the general lower performance of Slingshot pseudotime score, it is most likely due to the failure of the algorithm to identify a reliable root state, which is erroneously placed in a peripheric region right next to the monocyte branch rather than in the central multipotent basin (Fig. 6a, available as supplementary data at *Bioinformatics* online). This inconsistency results in principal curves that define an incorrect trajectory structure, and consequently in pseudotime scores that do not reliably recapitulate the potency gradient of several lineages, including the neutrophils (Fig. 6, available as supplementary data at *Bioinformatics* online).

As an additional analysis, we used as dynamic measure the pseudotime score of Monocle 3 (Cao *et al.* 2019), another very popular *top-down* trajectory reconstruction method that, unlike Slingshot, can only be used in supervised mode and forces the user to manually set the root. Here, the root of the genealogy was set within the cells that were sampled at culture Day 2 (Fig. 7, available as supplementary data at *Bioinformatics* online). Monocle 3 pseudotime score achieves a mean F_1_ score of 0.48 which, despite the manual root selection, is higher than Slingshot’s score but still lower than the scores of the two velocity measures. This suggests that the principal graph constructed by Monocle 3 is unsuitable to represent a complex genealogy with multiple lineages originating from the same multipotent subpopulation (Fig. 7, available as supplementary data at *Bioinformatics* online) and indicates that the specification of the root does not necessarily guarantee more accurate results if the algorithm is not optimal for the system at hand.

### 3.5 Performance benchmarking on simulated datasets

To further quantify the accuracy of the four methods that we compared in the murine hematopoiesis analysis, we used the dyngen simulation engine (Cannoodt *et al.* 2021) to generate simulated scRNA-seq datasets representing trajectories with known structures and directions. We then analyzed these simulated data with FIERCE, RNA velocity, Slingshot, and Monocle 3. Each dataset comprises 5000 cells, for which the expression values of ∼10 000 genes (both regulator and target genes) are modeled according to a simple bifurcating trajectory with a single branching point.

The accuracy was calculated by evaluating how well the expression values of cells, as ordered by each method, align with the known expression trends of key regulator genes (see Section 2 for details). Across a set of 10 simulated datasets, FIERCE achieves the highest median accuracy, followed by Monocle 3, Slingshot, and RNA velocity (Fig. 8, available as supplementary data at *Bioinformatics* online). Notably, FIERCE demonstrates similar accuracy across all simulated datasets, indicating higher stability in its performance.

We additionally assessed the effect of reduced dataset size on FIERCE performance. To this end, we progressively downsampled 5 of the 10 simulated trajectories by randomly selecting 2500, 1000, and 500 cells while preserving the relative abundance of the different trajectory sections. We then recomputed the accuracy of each method for each downsampled dataset. While the accuracy of Slingshot and Monocle 3 remained relatively stable across dataset sizes, the accuracy of both FIERCE and RNA velocity decreased as the number of cells was reduced. However, FIERCE maintained higher average accuracy than RNA velocity across downsampled datasets (Fig. 9, available as supplementary data at *Bioinformatics* online).

These results suggest that FIERCE, similarly to RNA velocity, benefits from sufficient sampling of the underlying dynamic process. Nevertheless, the higher accuracy of FIERCE across downsampled datasets indicates that inferring future cell dynamics through the rate of change of signaling entropy computed across a genome-wide PPI network, rather than through the rate of change of the raw expression signal, improves trajectory reconstruction under reduced sampling conditions.

## 4 Discussion

Among the various strategies to reconstruct dynamic and developmental trajectories (Chen *et al.* 2018, Saelens *et al.* 2019, Deconinck *et al.* 2021), a *bottom-up* approach holds the promise to reconstruct the final trajectory from cell differentiation potencies, which, as evidenced by recent studies, can be estimated from their transcriptional entropy, i.e. the level of dispersion of their gene expression signals across molecular pathways (Banerji *et al.* 2013, Guo *et al.* 2017, Teschendorff and Enver 2017, Chen *et al.* 2019). However, inferring the trajectory direction from the sole entropy requires the adoption of prior hypotheses on the expected dynamic behavior of cell entropies (Guo *et al.* 2017, Teschendorff and Enver 2017, Chen *et al.* 2019). This translates into the necessity of knowing in advance the expected direction of the final trajectory, which is the major drawback of traditional *top-down* methods (Teschendorff and Feinberg 2021).

Here, we introduce FIERCE, a novel *bottom-up* approach based on transcriptional entropy to reconstruct single-cell trajectories without the requirement of any preconceived assumptions on entropy dynamics. Specifically, FIERCE (i) reconstructs the topology of the differentiation landscape of cell populations in the signaling entropy space and (ii) predicts the future dynamics of such a landscape exclusively from the rate of change of the local entropy scores of single cells (i.e. from the velocity of the entropy). The rationale behind our tool is that the developmental evolution of a biological system emerges as a natural consequence of all the changes that the differentiation potency of all its cells undergoes over time. The velocity of the entropy constitutes the metric used by FIERCE to reconstruct the dynamics of cells in the potency space and synergizes the static information of signaling entropy with the dynamic information of RNA velocity.

By applying FIERCE to three scRNA-seq datasets representing diverse differentiation processes, we demonstrated its efficacy in reconstructing genealogies characterized by distinct structures and accurately predicting the future paths of cells independently from the underlying topology. To assess the additional insights provided by FIERCE in a completely unsupervised analysis context, we conducted a qualitative comparison of the vector field of the velocity of the entropy with both the RNA velocity vector field and the principal curves generated by Slingshot in the absence of manual specification of the root. FIERCE effectively reconstructed the complex branching MG developmental process without prior specification of either the genealogy’s origin or the expected location of branching events. In contrast, the vector field of RNA velocity exhibited no consistent pattern of arrow streams, likely due to the partitioned structure of the gene expression-based UMAP embedding. Similarly, Slingshot was unable to reconstruct the correct trajectory topology and consequently failed to compute a plausible pseudotime gradient, due to the erroneously estimated location of the root.

Leveraging the *ground-truth* fates determined by the clonal information provided by the LARRY protocol for the murine *in vitro* hematopoiesis, we quantitatively compared the efficacy of using the velocity of the entropy, RNA velocity, and Slingshot pseudotime score as inputs to predict cell differentiation fates through CellRank 2. Overall, FIERCE’s velocity of the entropy achieved similar accuracy compared to classic RNA velocity, but outperformed Slingshot pseudotime score. The velocity of the entropy and the RNA velocity accurately predicted the fates of multipotent progenitors, basophils, and monocytes, but both failed to identify lymphoid precursors as a separate lineage. RNA velocity performed better in predicting the fates of mast cells and neutrophils, but it barely identified the fate of megakaryocytes, which, instead, were identified by the velocity of the entropy. Despite the similar predictive accuracy of the two velocity measures, the velocity of the entropy provided a more reliable visual depiction of this differentiation system, particularly for the lymphoid lineage and the embedding region that includes basophils, mast cells, and megakaryocytes. Slingshot was again unable to correctly identify the position of the root, and thus constructed incorrect principal curves that resulted in unreliable pseudotime scores.

As evidenced by our analyses, even one of the top-performing algorithms based on the traditional *top-down* approach exhibits serious limitations without external supervision. This is due to the inherent nature of the *top-down* workflow: first, the structure of the trajectory is constructed based on raw expression data, and then the pseudotime score (acting as a proxy of the differentiation potency of cells) is computed *a posteriori* based on the projection of cells onto this structure (Teschendorff and Feinberg 2021). If the trajectory structure is not properly constructed (e.g. due to uncertainty in the position of the root), then all pseudotime scores will be unreliable, and the potencies of cells will not be correctly estimated. The *bottom-up* approach employed by FIERCE offers the fundamental advantage to reconstruct the macroscopic dynamics of the entire cell population by directly predicting the microscopic changes in the differentiation potencies of individual cells. These potencies are estimated *a priori* and in a completely unbiased manner using their signaling entropy scores. Thus, the structure and direction of the trajectory naturally emerge from the dynamics of cell potencies, rather than the other way around. This crucial difference enabled FIERCE to construct reliable and robust trajectories for all tested datasets, irrespective of the population structure or any prior assumptions about the direction of the dynamic process. Moreover, it is worth noting that the vector field constructed by FIERCE for the hematopoiesis dataset is consistent with a recent reconstruction of the hematopoiesis process that places progenitors of basophils, mast cells, megakaryocytes, and erythrocytes into a separate branch (Tusi *et al.* 2018). This detail, which is not apparent in the vector field constructed from RNA velocity, indicates that the additional information provided by signaling entropy (i.e. the distribution of the gene expression signal of cells over the genome-wide PPI network) enables FIERCE not only to reconstruct an accurate representation of the genealogy and infer the dynamic changes in the cell potency landscape but also to gain additional insights that can help clarify the details of the most complex differentiation systems. Finally, the analysis of simulated scRNA-seq datasets demonstrated FIERCE’s superior performance in accurately reconstructing complex cellular trajectories, in terms of accuracy and stability.

Although downsampling analyses indicate that FIERCE performance is affected by the number of cells available to reconstruct the underlying dynamic process, FIERCE maintained higher accuracy than RNA velocity across the downsampled datasets, suggesting that the signaling-entropy representation partially improves robustness to reduced cell number. We therefore recommend applying FIERCE to datasets in which the trajectory is sufficiently sampled, with adequate representation of intermediate states and terminal branches. The required number of cells is expected to depend on the complexity of the biological process, the number of branches or cell states, and the abundance of transitional populations. Since FIERCE incorporates RNA velocity estimates, its performance is also expected to be influenced by sequencing depth. RNA velocity relies on accurate quantification of spliced and unspliced molecules, and recent benchmarking has shown that velocity predictions can change substantially when reads are downsampled (Ancheta *et al.* 2026). Users should therefore apply FIERCE to datasets that pass standard scRNA-seq quality control, have sufficient sequencing depth for the platform used, and show adequate recovery of spliced and unspliced molecules.

As scRNA-seq technologies are increasingly applied to biological systems of growing complexity, there is a continuous demand for entirely unsupervised methods capable of reconstructing comprehensive and detailed depictions of the differentiation potency landscape of these systems with minimal input from researchers. Based on the results presented in this study, we believe that FIERCE could serve as a robust starting point for the development of a new generation of algorithms designed to achieve this goal through a fully *bottom-up* approach inspired by the principles of statistical mechanics (MacArthur and Lemischka 2013, Teschendorff and Feinberg 2021). Such a change in perspective is particularly necessary given the current emphasis on developing computational methods capable of extracting dynamic information from the diverse types of data profiled by modern single-cell sequencing technologies. For example, new methods are being developed to reconstruct dynamic trajectories from single-cell epigenomic data, either alone or in combination with transcriptomic data (Deconinck *et al.* 2021, Tedesco *et al.* 2022, Li *et al.* 2023). In this context, it is crucial to understand how the interplay between the transcriptome and various epigenomic factors determines both the current differentiation potencies of cells and how these potencies will change in the near future (MacArthur and Lemischka 2013). Here, we have demonstrated the potential of the *bottom-up* approach when applied to transcriptomic data. However, we envision that extending this concept to other omics data types will facilitate the development of novel, composite computational pipelines that can harness the full complexity of single-cell molecular profiles to reconstruct the evolution of tissues and organs under both physiological and pathological conditions.

## Author contributions

Luca Calderoni (Conceptualization [lead], Data curation [equal], Formal analysis [lead], Methodology [lead], Software [lead], Visualization [lead], Writing—original draft [lead]), Oriana Romano (Data curation [supporting], Writing—review & editing [equal]), Francesco Grandi (Data curation [equal], Software [lead]), Mattia Forcato (Conceptualization [equal], Funding acquisition [equal], Supervision [equal], Writing—original draft [equal], Writing—review & editing [equal]), and Silvio Bicciato (Funding acquisition [equal], Supervision [supporting], Visualization [equal], Writing—original draft [equal], Writing—review & editing [equal])

## Supplementary Material

btag516_Supplementary_Data

## Data Availability

The pancreas endocrinogenesis dataset was retrieved from the scVelo Python package (v0.2.4). The UMI counts and fastq files of the murine mammary gland development dataset were downloaded from Gene Expression Omnibus (GEO, GSE111113) and the Sequence Read Archive (SRA, SRP133477), respectively. The fastq files of the *in vitro* murine hematopoiesis dataset were downloaded from Sequence Read Archive (SRX7199569).

## Code availability

FIERCE is written in R language and is released under a GPL License. The package can be downloaded from GitHub (https://github.com/bicciatolab/FIERCE) along with an installation guide and an application tutorial. The version used in all the analyses of this study is available on figshare (https://doi.org/10.6084/m9.figshare.32188575).
