# Supplementary material for: FIERCE: reconstructing dynamic trajectories from the differentiation potency of single cells
Source: Bioinformatics. 2026 Jul 13;42(7):btag516. doi: 10.1093/bioinformatics/btag516 (PMC13415460; doi:10.1093/bioinformatics/btag516)
Supplement: btag516_Supplementary_Data [file btag516_supplementary_data.docx]

Supplementary Table 1: Cell type composition of the pancreas endocrinogenesis dataset.

| **Cell type** | **Number of cells** |
| --- | --- |
| DUCTAL CELLS | 916 |
| *Ngn3*^-^ ENDOCRINE PROGENITORS | 262 |
| *Ngn3^+^* ENDOCRINE PROGENITORS | 642 |
| PRE-ENDOCRINE CELLS | 592 |
| ALPHA CELLS | 481 |
| BETA CELLS | 591 |
| DELTA CELLS | 70 |
| EPSILON CELLS | 142 |
| **Total** | **3696** |

Supplementary Table 2: Type and sampling age of the cells analysed in the reconstruction of mouse mammary gland development.

| **Cell type** | **Embryonic day 16** | **Embryonic day 18** | **Postnatal day 4** | **Adult** | **Total** |
| --- | --- | --- | --- | --- | --- |
| EMBRYONIC EPITHELIAL CELLS | 692 | 606 | 7 | 0 | 1305 |
| LUMINAL PROGENITORS | 1 | 41 | 431 | 63 | 536 |
| ALVEOLAR PRECURSOR CELLS | 0 | 144 | 312 | 693 | 1149 |
| MATURE LUMINAL CELLS | 0 | 4 | 321 | 1022 | 1347 |
| BASAL CELLS | 5 | 320 | 213 | 293 | 831 |
| **Total** | **698** | **1115** | **1284** | **2071** | **5168** |

**Supplementary Table 3**: Cells of the *in vitro* murine hematopoiesis dataset that were successfully assigned to a specific clone by the LARRY protocol, subdivided by cell type and sampling time point.

| **Cell type** | **Day 2** | **Day 4** | **Day 6** | **Total** |
| --- | --- | --- | --- | --- |
| MULTIPOTENT CELLS | 3148 | 8402 | 11 169 | 22 719 |
| LYMPHOID PRECURSORS | 14 | 101 | 88 | 203 |
| ERYTHROID PROGENITORS | 9 | 71 | 282 | 362 |
| MEGAKARYOCYTES | 1 | 291 | 734 | 1026 |
| MAST CELLS | 3 | 245 | 1158 | 1406 |
| BASOPHILS | 11 | 1343 | 4139 | 5493 |
| EOSINOPHILS | 0 | 36 | 132 | 168 |
| NEUTROPHILS | 15 | 2453 | 5959 | 8427 |
| MONOCYTES | 29 | 1996 | 5961 | 7986 |
| MIGRATORY DCs | 7 | 17 | 40 | 64 |
| PLASMACYTOID DCs | 0 | 30 | 17 | 47 |
| **Total** | **3237** | **14** **985** | **29** **679** | **47** **901** |

**Supplementary Table 4**: *Ground-truth* fates of the 21 726 cells of the hematopoiesis dataset that were successfully assigned to a single differentiation lineage.

| **Cell fate** | **Day 2** | **Day 4** | **Day 6** | **Total** |
| --- | --- | --- | --- | --- |
| MULTIPOTENT CELLS | 484 | 1878 | 4228 | 6590 |
| LYMPHOID PRECURSORS | 81 | 113 | 121 | 315 |
| MEGAKARYOCYTES | 51 | 267 | 511 | 829 |
| MAST CELLS | 8 | 55 | 185 | 248 |
| BASOPHILS | 126 | 651 | 1777 | 2554 |
| NEUTROPHILS | 320 | 1747 | 3018 | 5085 |
| MONOCYTES | 415 | 1738 | 3952 | 6105 |
| **Total** | **1485** | **6449** | **13** **792** | **21** **726** |

**Supplementary Table 5:** F_1_ scores, precision, and recall of the differentiation fates predicted by CellRank 2 for the cells of the *in vitro* murine hematopoiesis dataset based on the velocity of the entropy, the RNA velocity, and the pseudotime score of Slingshot.

|  | **Velocity of the entropy** | | | **RNA velocity** | | | **Pseudotime** | | |
| --- | --- | --- | --- | --- | --- | --- | --- | --- | --- |
| **Differentiation fate** | *F1* | *precision* | *recall* | *F1* | *precision* | *recall* | *F1* | *precision* | *recall* |
| MULTIPOTENT CELLS | 0.64 | 0.48 | 0.95 | 0.65 | 0.53 | 0.84 | 0.02 | 0.11 | 0.01 |
| LYMPHOID PRECURSORS | 0 | 0 | 0 | 0 | 0 | 0 | 0.05 | 0.03 | 0.93 |
| MEGAKARYOCYTES | 0.50 | 0.99 | 0.34 | 0.16 | 1 | 0.09 | 0.63 | 0.50 | 0.87 |
| MAST CELLS | 0.55 | 0.40 | 0.88 | 0.71 | 0.97 | 0.56 | 0.74 | 0.73 | 0.74 |
| BASOPHILS | 0.80 | 0.96 | 0.69 | 0.78 | 0.78 | 0.78 | 0.81 | 0.80 | 0.82 |
| NEUTROPHILS | 0.36 | 1 | 0.22 | 0.68 | 0.81 | 0.58 | 0 | 0 | 0 |
| MONOCYTES | 0.80 | 0.90 | 0.73 | 0.77 | 0.88 | 0.69 | 0.84 | 0.94 | 0.77 |
| **Mean** | **0.52** | **0.68** | **0.54** | **0.54** | **0.71** | **0.51** | **0.44** | **0.44** | **0.59** |


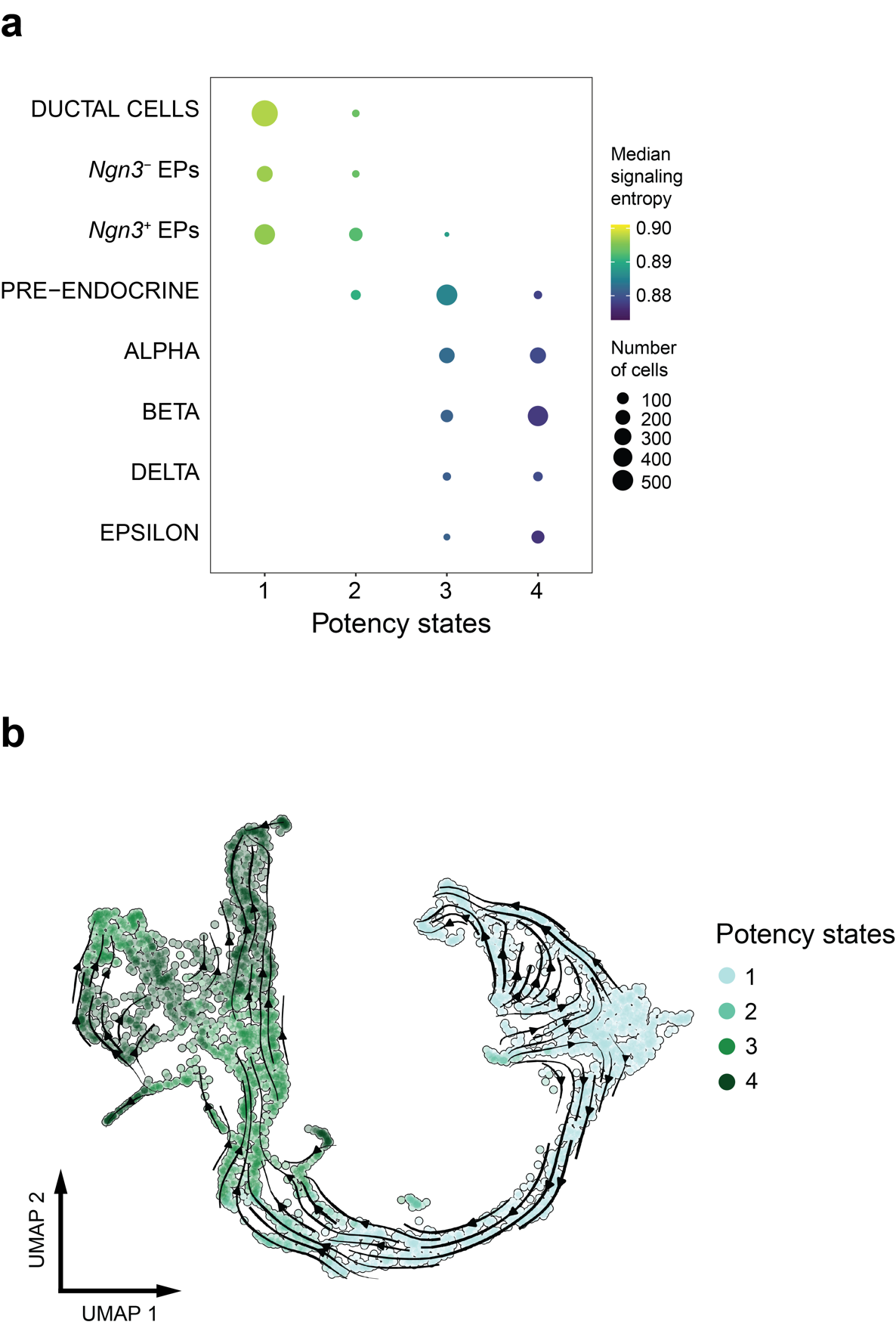


**Supplementary Figure 1. Murine pancreas endocrinogenesis.**

Distribution of potency states in the murine pancreas endocrinogenesis dataset (**a**) across cell types and (**b**) on the streamplot generated by FIERCE. The potency states are numbered according to their mean signaling entropy score in decreasing order, i.e., states with lower numbers have higher mean scores.


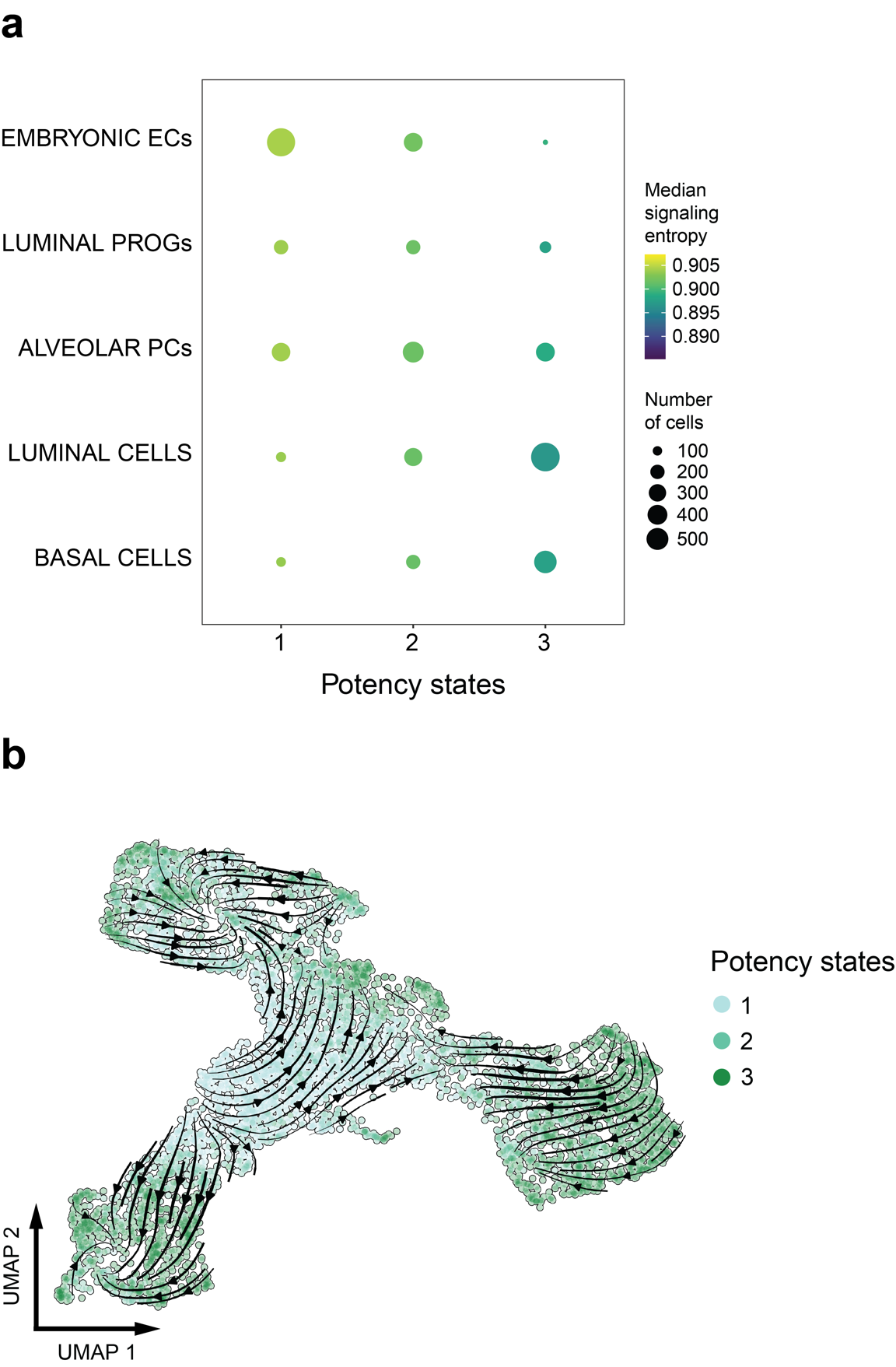


**Supplementary Figure 2. Murine mammary gland development.**

Distribution of the potency states in the mammary gland development dataset (**a**) across cell types (**b**) and on the streamplot generated by FIERCE. The potency states are numbered according to their mean signaling entropy score in decreasing order, indicating that states with lower numbers have higher mean scores.

**
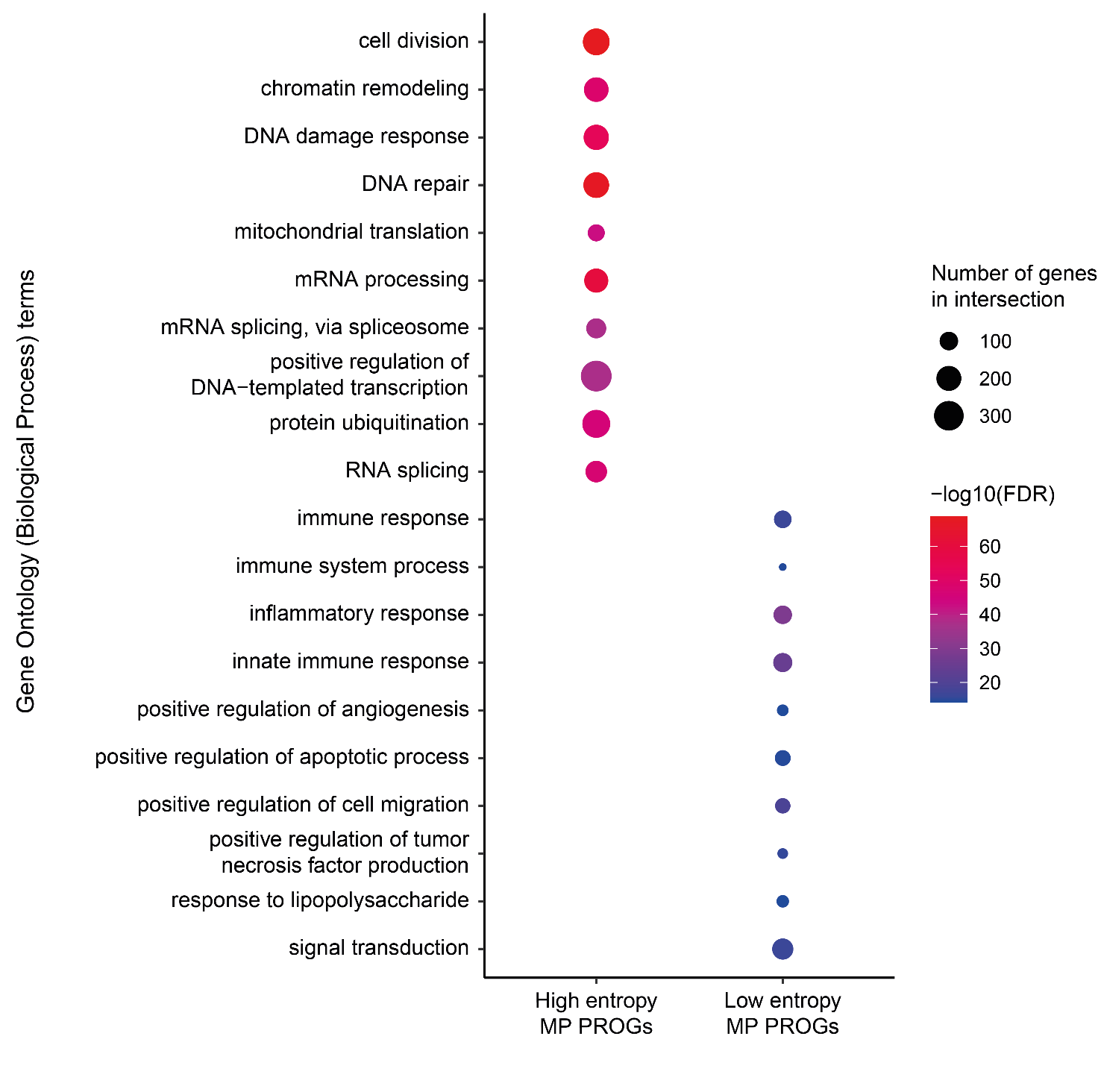
**

**Supplementary Figure 3. Functional enrichment analysis of multipotent progenitors in the *in vitro* murine hematopoiesis dataset.**

Dotplot showing the top 10 Gene Ontology (Biological Process) terms significantly enriched in upregulated genes in either high entropy (top 25%) or low entropy (bottom 25%) multipotent progenitor cells. The size of dots is proportional to the intersection between the upregulated genes in either high or low entropy progenitors and the genes included in each term. The colour of dots represents the negative log_10_(FDR) of each term. MP PROGs: multipotent progenitors.

**
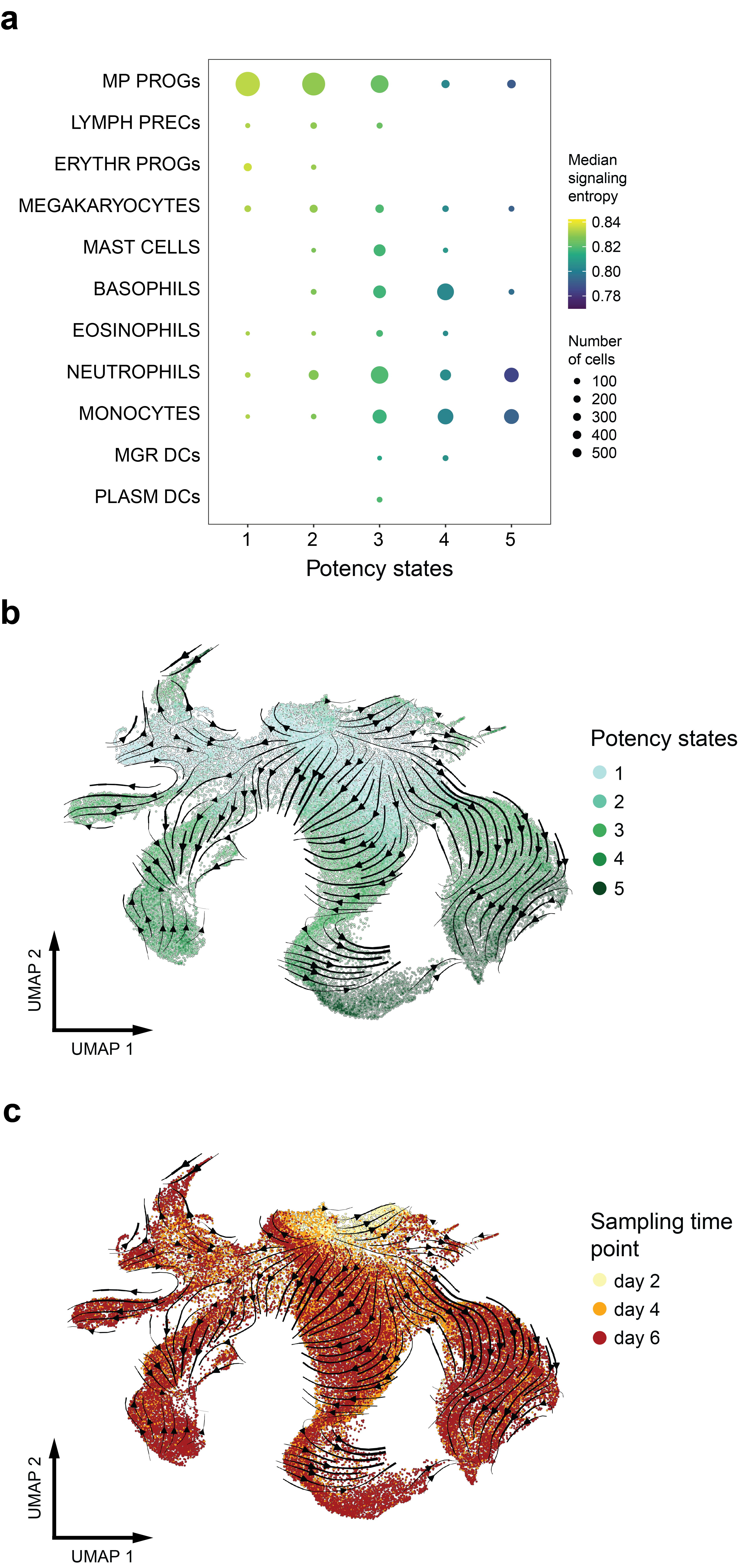
**

**Supplementary Figure 4. *In vitro* murine hematopoiesis.**

Distribution of the potency states in the *in vitro* murine hematopoiesis dataset (**a**) across cell types and (**b**) on the streamplot generated by FIERCE. The potency states are numbered according to their mean signaling entropy score in decreasing order, indicating that states with lower numbers have higher mean scores. **c**) Distribution of the sampling time points (days in culture) on the streamplot generated by FIERCE. MP PROGs: multipotent progenitors; LYMPH PRECs: lymphoid precursors; ERYTHR PROGs: erythroid progenitors; MGR DCs: migratory dendritic cells; PLASM DCs: plasmacytoid dendritic cells.

**
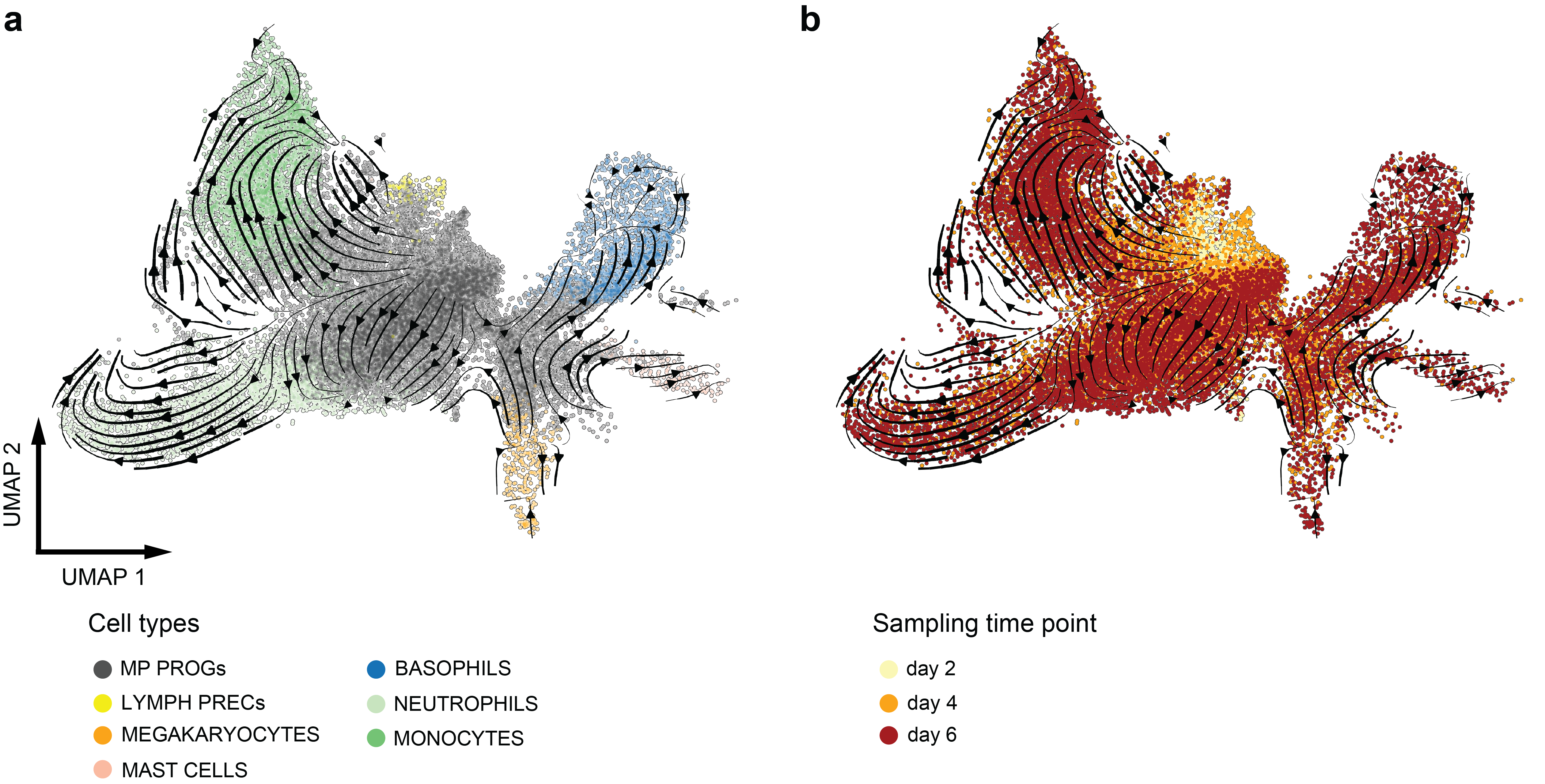
**

**Supplementary Figure 5. RNA velocity vector field built by scVelo on a UMAP embedding for the *in vitro* hematopoiesis dataset.**

Cells are coloured according to (**a**) cell type labels and (**b**) sampling time points. Only the cells included in the CellRank 2 analysis are shown. MP PROGs: multipotent progenitors; LYMPH PRECs: lymphoid precursors.

**
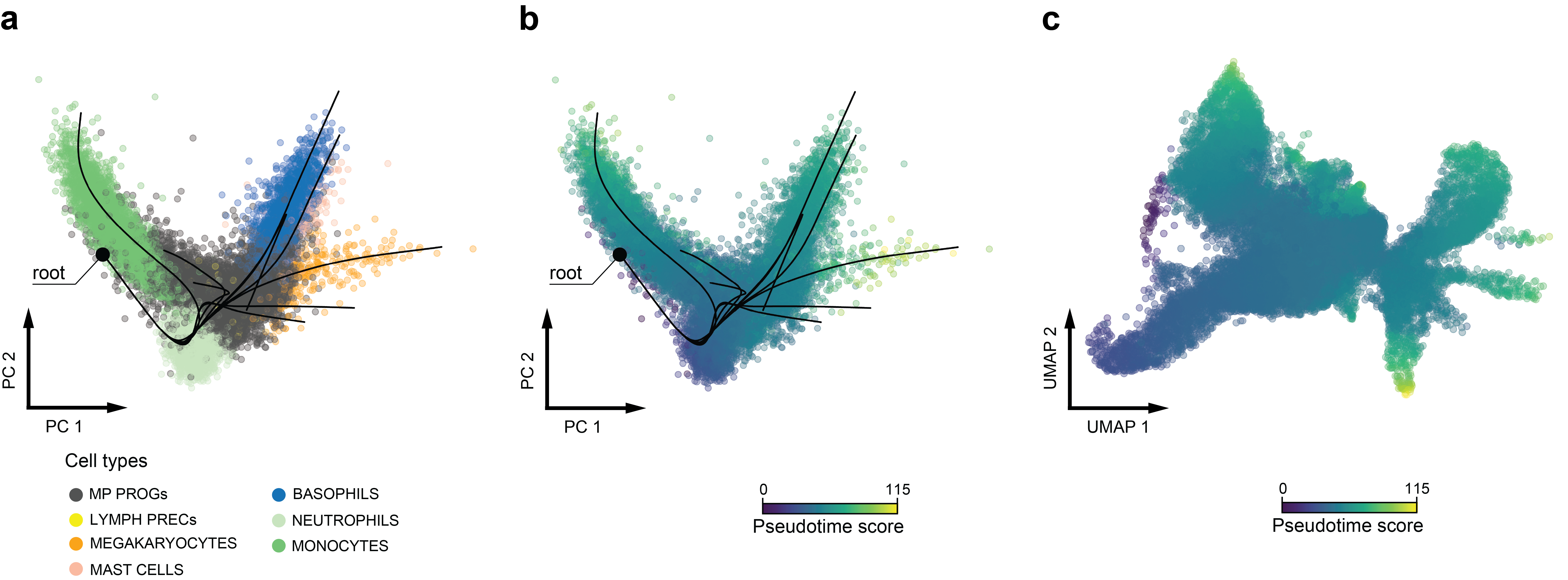
**

**Supplementary Figure 6. Principal curves and pseudotime scores computed by Slingshot for the *in vitro* hematopoiesis dataset.**

**a-b)** Principal curves constructed by Slingshot on the space defined by the first two principal components. Cells are coloured according to (**a**) cell type labels and (**b**) pseudotime score. Only the cells included in the CellRank 2 analysis are shown. **c)** UMAP embedding with cells coloured according to their Slingshot pseudotime scores. MP PROGs: multipotent progenitors; LYMPH PRECs: lymphoid precursors.


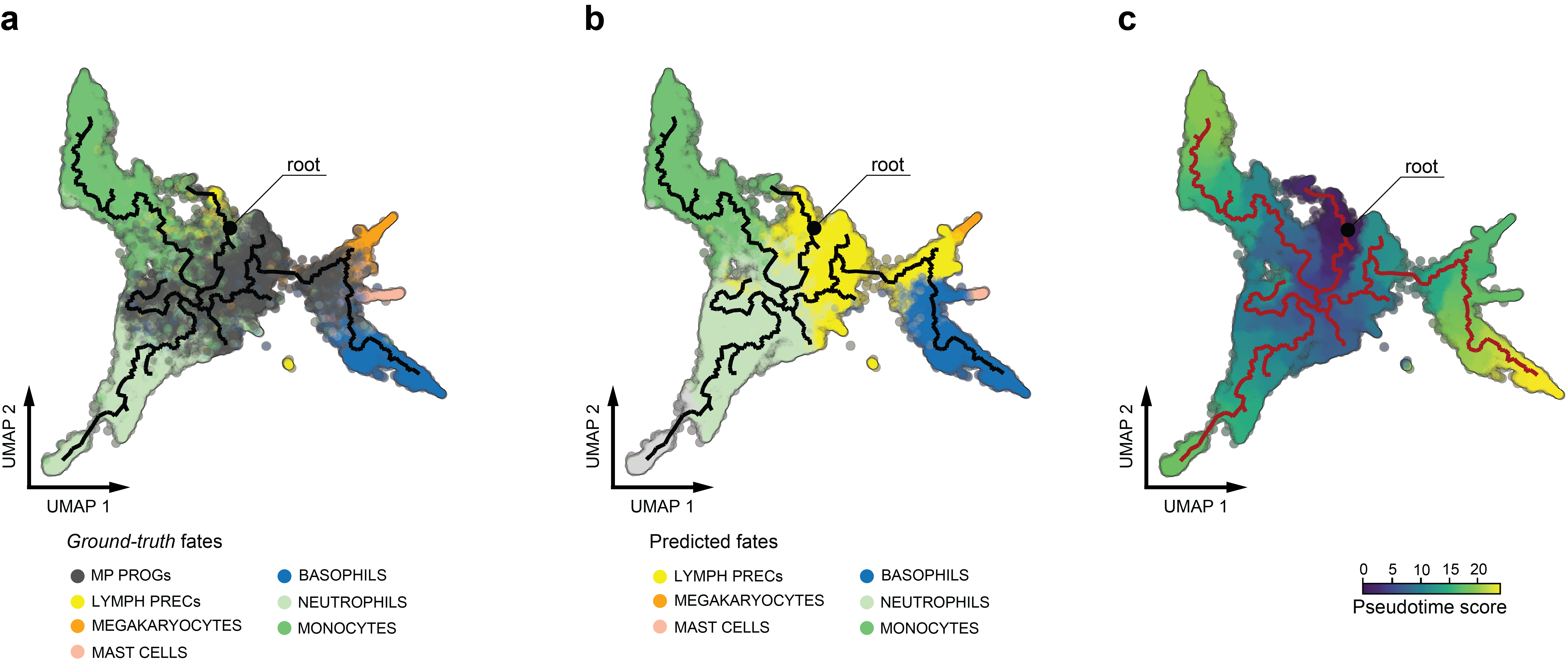


**Supplementary Figure 7. Principal graph and pseudotime scores computed by Monocle 3 for the *in vitro* hematopoiesis dataset.**

The principal graph was built on a UMAP embedding. Cells are coloured according to (**a**) the *ground-truth* fates evinced from the clonal information provided by the LARRY protocol, (**b**) the fates predicted by the CellRank 2 algorithm based on Monocle 3 pseudotime scores and (**c**) the pseudotime scores. Only the cells included in the CellRank 2 analysis are shown. MP PROGs: multipotent progenitors; LYMPH PRECs: lymphoid precursors.

**
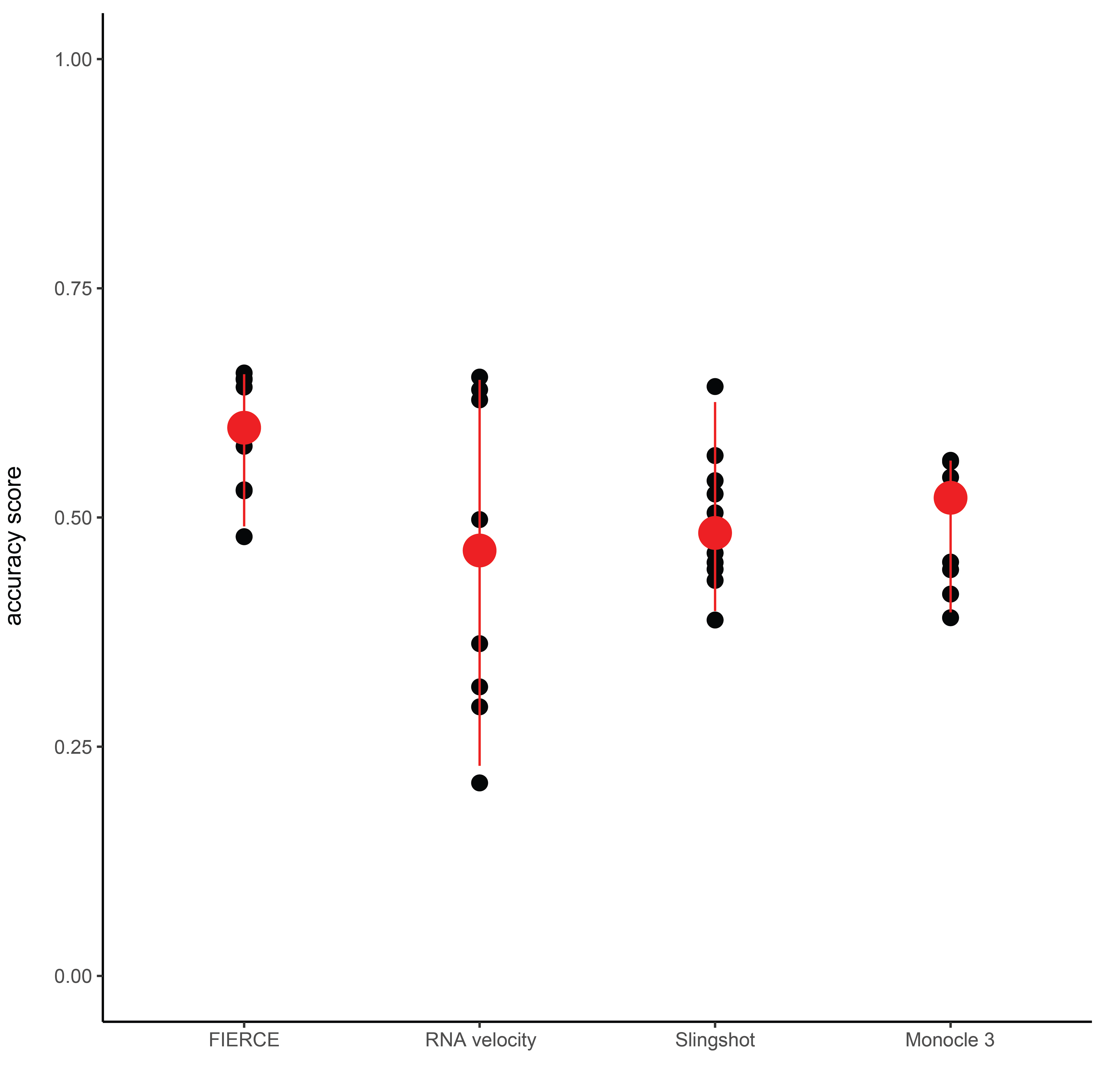
**

**Supplementary Figure 8. Accuracy scores achieved by FIERCE, RNA velocity, Slingshot and Monocle 3 in the reconstruction of 10 simulated trajectories with known structure and direction.**

The red dots represent median values, while the red lines represent the interquartile ranges.

**
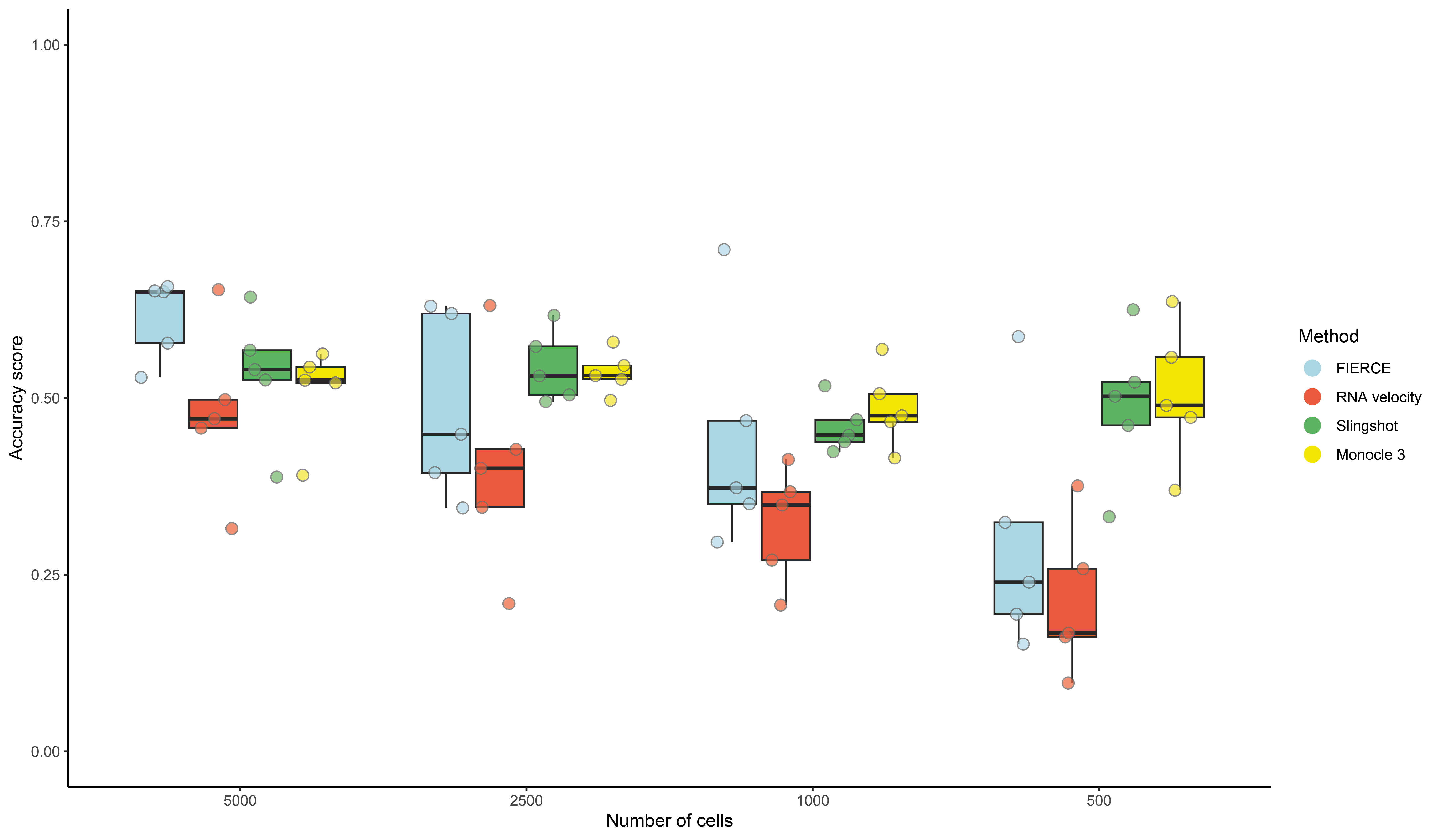
**

**Supplementary Figure 9. Boxplot of the accuracy scores achieved by FIERCE, RNA velocity, Slingshot, and Monocle 3 in the reconstruction of 5 simulated trajectories at progressively decreasing dataset sizes.** The boxes represent the median values and the interquartile ranges, while the whiskers represent the total ranges (excluding outliers).
